# Molecular signatures of adaptive introgression and selection in contact zones of closely related pine species (*Pinus* genus)

**DOI:** 10.1186/s12870-025-07490-x

**Published:** 2025-10-21

**Authors:** Sebastian Szczepański, Bartosz Łabiszak, Witold Wachowiak

**Affiliations:** 1 Department of Plant Ecology and Environmental Protection, Institute of Environmental Biology, Adam Mickiewicz University in Poznań, Uniwersytetu Poznańskiego 6, Poznań, 61-614 Poland; 2 Department of Genetics and Environmental Interactions, Institute of Dendrology, Polish Academy of Sciences, Parkowa 5, Kórnik, 62-035 Poland

**Keywords:** Hybridization, Adaptation, Hybrid zones, Introgression, Genomic diversity, Adaptive evolution

## Abstract

**Background:**

Natural hybridization plays a key role in shaping genetic diversity, local adaptation, and the dynamics of speciation through interspecific gene flow. Hybrid zones serve as valuable natural systems for studying these processes. In this research, we used genotypic data at thousands of nuclear SNPs to investigate genomic outcomes of hybridization and selection across three contact zones of closely related pine species including Scots pine (*Pinus sylvestris* L.) and dwarf mountain pine (*P. mugo* T.). Reference allopatric stands of parental species were used to assess introgression dynamics.

**Results:**

Individuals from the hybrid zones showed distinct genetic ancestry patterns and were assigned to groups including putative pure species, first-generation hybrids, and advanced backcrosses. Genotypes of the majority of hybrids were shifted towards *P. mugo* ancestry. Most outlier loci were shared across all sympatric populations, although some were specific to individual contact zones. The identified outliers were mainly associated with regulatory biological processes related to phosphorylation, proteolysis, and transmembrane transport. Signatures of local adaptation varied in different genetic classes in contact zones and they were strongest in pure *P. sylvestris* and hybrids with a majority of *P. sylvestris* ancestry. The pattern suggests that it may be driven by adaptation to peat bog habitats situated outside the species’ core ecological niche.

**Conclusions:**

Our findings indicate strong selective pressure acting on multiple genes in groups of hybrids and pure *Pinus sylvestris* individuals across all studied hybrid zones. In contrast, the weaker signal of selection observed in individuals with *P. mugo* ancestry suggests that relict populations of this species, which historically spread across postglacial peat bogs, were pre-adapted to such environments. While several outlier loci were shared across different contact zones, others were unique for one of them, indicating that local environmental pressures and adaptive introgression shape the genomic composition of the populations. These results highlight the role of hybridization in generating adaptive diversity and emphasize the evolutionary significance of hybrid zones in pines.

**Supplementary Information:**

The online version contains supplementary material available at 10.1186/s12870-025-07490-x.

## Background

Natural hybridization is a significant evolutionary process that shapes the genetic architecture and adaptive potential of populations. It can promote speciation by generating novel genetic combinations that are exposed to natural selection [1]. The exchange of genetic material between species may lead to new phenotypic traits, some of which may confer fitness advantages under changing environmental conditions [2]. Understanding the dynamics of hybridization provides valuable insights into mechanisms of adaptive introgression, including how it generates novel evolutionary trajectories and how natural selection influences the fitness of hybrid individuals.

In recent years, numerous cases of hybridization have been documented in both animal [3–6] and plant systems [7–10]. Plants, in particular, show a high propensity for hybridization, driven by factors such as overlapping reproductive strategies (e.g. flowering phenologies) and generally weaker reproductive barriers compared to animals [11]. Despite this, only a limited number of plant groups have been studied in the context of hybrid zones, including model species from *Helianthus* [12], *Iris* [13], *Mimulus* [14], and *Senecio* [15] genus. In plants, hybridization can lead to a range of outcomes – from transient gene exchange to the formation of stable hybrid lineages or even new species. These outcomes are often highly context-dependent, shaped by environmental heterogeneity, life history traits, and the genomic architecture of reproductive barriers, meaning the genetic basis and distribution of loci underlying reproductive isolation. Thus, expanding the study of hybrid zones – especially in long-lived and ecologically dominant taxa such as forest trees – is essential for advancing our understanding of how hybridization influences genetic structure, fitness, and long-term population viability in plants [16, 17]. Forest trees, particularly closely related species, offer compelling systems for studying natural hybridization, as seen in oaks [18–21], poplars [22–24], and birches [25–27]. In conifers, hybridization has been documented for instance in spruces [28–30], firs [31, 32], junipers [33, 34], yews [35] and pines [36–41]. Trees are particularly well-suited for studying hybridization due to their long generation times, large effective population sizes, and extensive pollen and seed dispersal, which allow introgressed alleles to persist and spread across landscapes [42]. Their broad ecological niches expose hybrid populations to diverse selection pressures, making them ideal for investigating how gene flow interacts with local adaptation and shapes evolutionary trajectories [43]. These processes are especially significant in forest ecosystems, where interspecific gene flow can influence traits related to growth, resistance to environmental stress, and reproductive success, resulting in complex genetic structures and adaptive variation [44].

Pines (*Pinus* spp.) are evergreen conifers of the *Pinaceae* family, widespread across the Northern Hemisphere [45]. They exhibit diverse and dynamic patterns of hybridization facilitated by overlapping geographic distributions, similar reproductive biology, and shared ecological strategies [46–54]. Within this genus, Scots pine (*Pinus sylvestris* L.) and taxa from the *Pinus mugo* complex, including dwarf mountain pine (*P. mugo* Turra) represent a particularly interesting system for investigating taxonomic relationships, hybridization, and evolutionary processes. These taxa are primarily allopatric across Europe, occupying a broad range of habitats from alpine and subalpine zones to peat bogs and lowland forests [55]. Taxonomic relationships within the *Pinus mugo* complex (including among others such taxa as *P. mugo*,* P. uliginosa* and *P. uncinata*) are intricate, with ongoing debates regarding species delineation and genetic differentiation. Genetic and phylogeographic studies have revealed that the *Pinus mugo* complex history was shaped by historical events, such as glaciations, and contemporary gene flow [53–59]. Hybridization between *P. sylvestris* and members of the *P. mugo* complex has been repeatedly documented in natural populations and experimental settings [52, 60, 61]. Isozyme analyses of hybrid swarms in Slovakia revealed that admixed individuals show stronger affinity to *P. mugo* than to *P. sylvestris* [62], consistent with earlier reports of asymmetric introgression favoring *P. mugo* ancestry [63]. Controlled reciprocal crosses confirmed interfertility of the taxa but also demonstrated partial reproductive barriers, such as reduced seed viability and a higher proportion of empty seeds in certain cross directions [64, 65]. These findings point to incomplete pre- and post-zygotic isolation, with gene flow occurring despite constraints imposed by phenology and pollen–pistil interactions. Molecular marker studies further supported the presence of extensive hybridization in contact zones, with introgression restricted to sympatric stands and absent from allopatric populations [51, 54]. Together, these results indicate that hybridization between *P. sylvestris* and the *P. mugo* complex is widespread, asymmetrical, and shaped by both ecological factors and partial reproductive barriers, providing an important context for investigating the genomic basis of introgression and adaptation. However, critical gaps remain in our understanding of the genetic interactions and adaptive processes shaping these closely related taxa.

The main objectives of this study were to investigate the role of interspecific gene flow and natural selection in shaping genetic diversity and evolutionary trajectories across several contact zones between *Pinus sylvestris* and *P. mugo*, each differing in environmental context. We applied high-throughput genotyping of thousands of nuclear SNP polymorphisms across more than 1,500 individuals sampled from both allopatric populations and hybrid zones. Specifically, we aimed to (i) characterize patterns of interspecific gene flow, (ii) assess the influence of hybridization on genetic structure in contrasting environments, and (iii) identify genomic regions that undergo introgression and loci under selection. We hypothesized that adaptive introgression facilitates the transfer of beneficial alleles between taxa, with positive selection acting for loci associated with local environmental adaptations. Additionally, we expected that similar environments would promote the selection on the same genetic variants, whereas distinct habitats would exert divergent selective pressures on different alleles.

The ecological settings in which the two species co-occur provide important context for these hypotheses. *P. sylvestris* typically occupies drier, mineral-rich soils at lower elevations, whereas *P. mugo* is more tolerant of water-logging, poor aeration, and nutrient limitation, thriving in peat bogs and mountain habitats [55–57]. In contact zones, these contrasting ecological preferences overlap, resulting in mosaic habitats where the species and their hybrids coexist under variable microenvironmental conditions. This environmental heterogeneity is likely to shape both the direction and the fitness consequences of introgression, by favoring alleles conferring tolerance to locally limiting factors such as excess water or nutrient scarcity. Therefore, we hypothesize that adaptive introgression facilitates the persistence of *P. sylvestris* in marginal bog habitats through the acquisition of stress-tolerance alleles from *P. mugo*, whereas *P. mugo*-like hybrids maintain high fitness due to their preadaptation to such environments. We further predict that introgressed alleles associated with physiological stress responses will be overrepresented in hybrid genomes contributing to the establishment of hybrids in challenging microhabitats and thereby shaping the evolutionary trajectories of both taxa in their contact zones.

To our knowledge, this is one of the most extensive genomic investigations of hybridization in *Pinus*, integrating high-resolution nuclear SNP data across multiple contact zones and environmental contexts. This approach allows us to explore how ecological and geographic variation jointly shape patterns of gene flow and selection in hybridizing conifer taxa.

## Materials and methods

### Studied populations and sampling

**Fig. 1:**
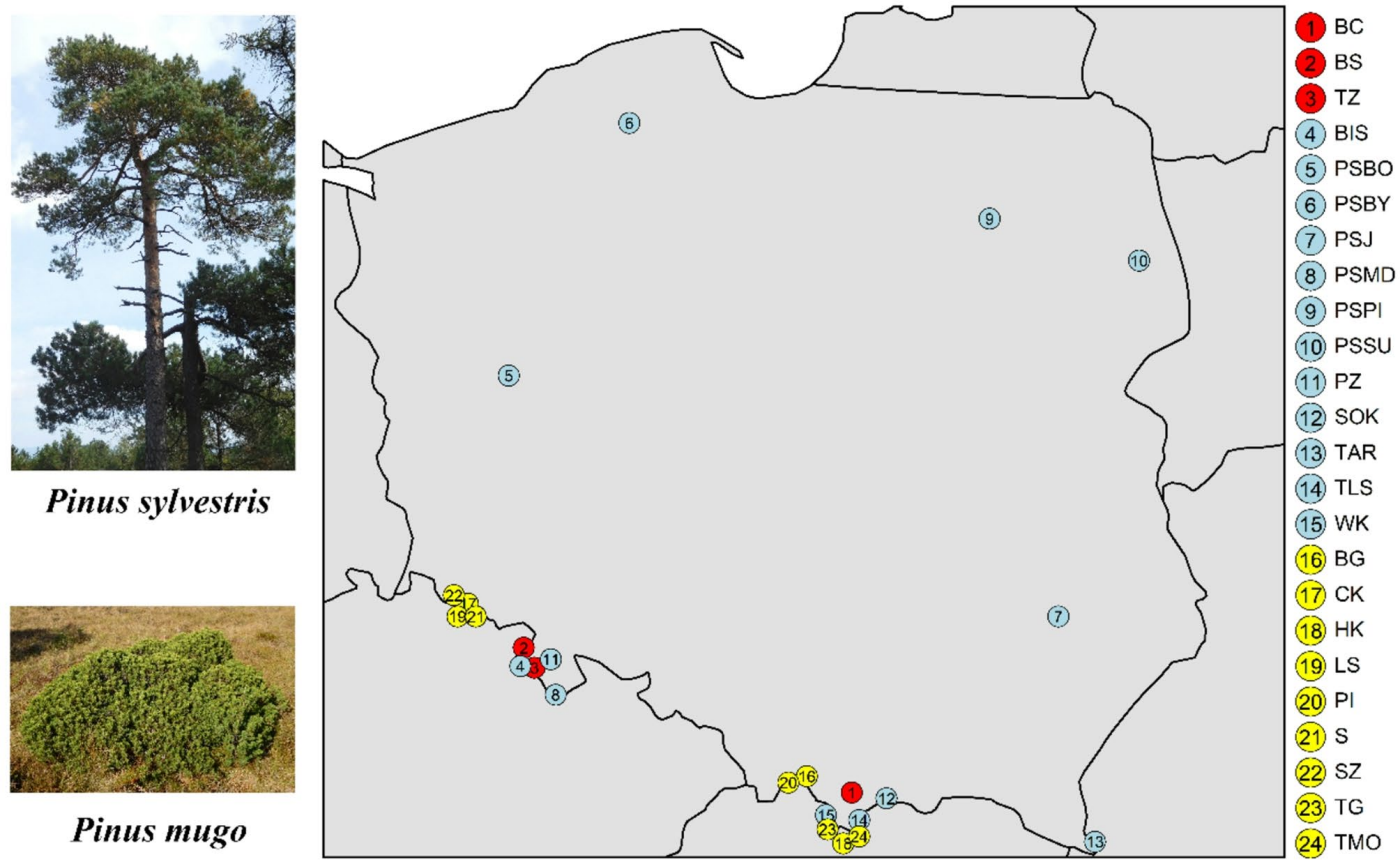
Geographic distribution of the analyzed populations in Poland. Red circles indicate populations from *P. sylvestris* - *P. mugo* contact zones. Blue symbols represent reference populations of *Pinus sylvestris*, while yellow symbols denote reference stands of *P. mugo*. Numbers correspond to population codes listed in Supplementary Table S1

**Fig. 2:**
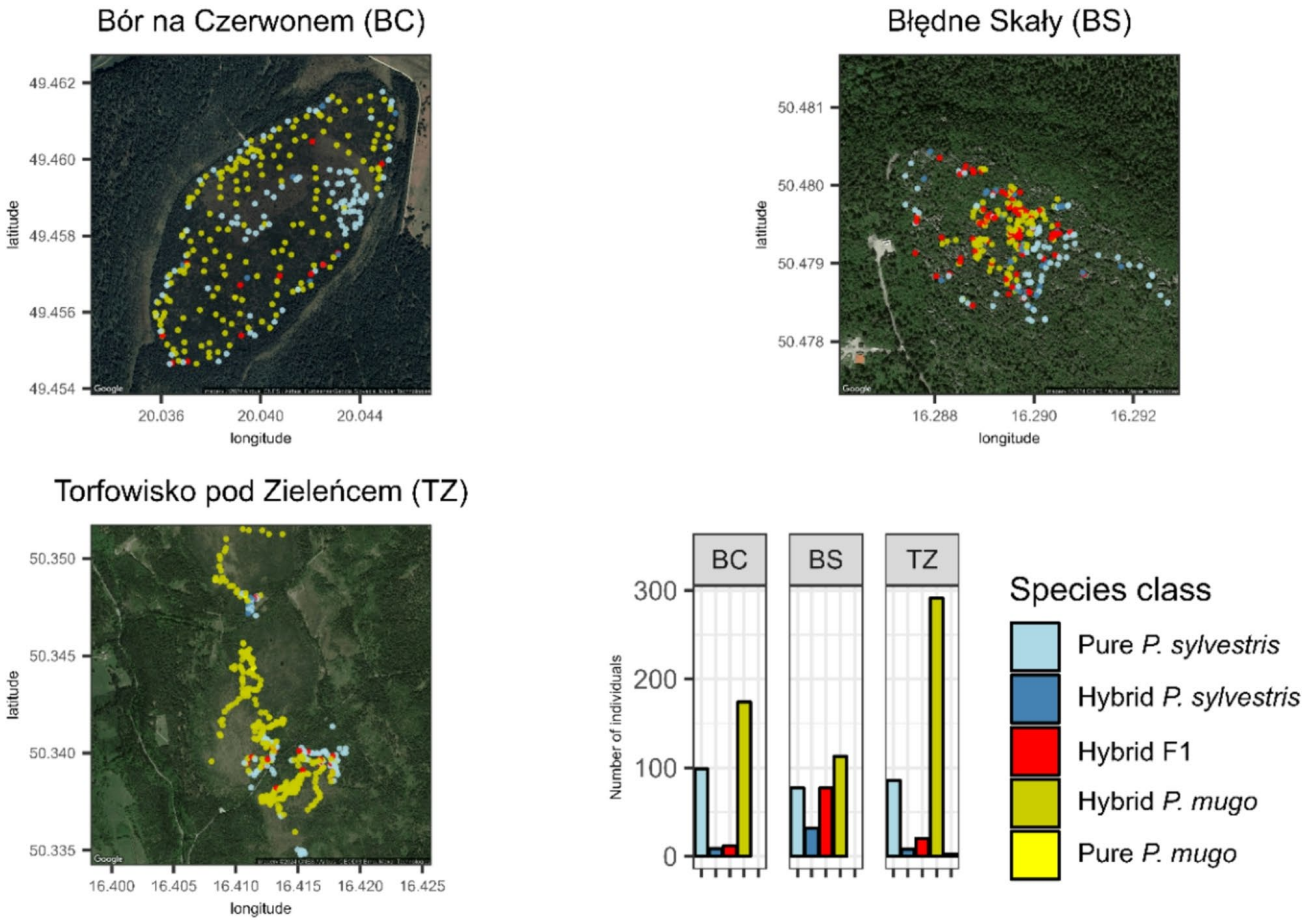
Spatial distribution and genetic classification of pine individuals across three hybrid zones: Bór na Czerwonem (BC), Błędne Skały (BS), and Torfowisko pod Zieleńcem (TZ) located in Poland. Maps show the geographic position of sampled individuals colored by species class as inferred from genetic clustering: pure *Pinus sylvestris* (light blue), hybrid *P. sylvestris* (dark blue), F1 hybrids (red), hybrid *P. mugo* (dark yellow), and pure *P. mugo* (yellow). Bar plots indicate the number of individuals assigned to each class per site, illustrating differences in hybrid composition and taxonomic structure among zones

A total of 1,558 trees were genotyped from 24 populations, comprising three hybrid zones and 21 reference populations. The reference populations included 12 Scots pine (*Pinus sylvestris*, PS) and 9 dwarf mountain pine (*P. mugo*, PM) populations (Fig. 1). The hybrid zones investigated in this study were located in southern and southwestern Poland and included Bór na Czerwonem (BC), Błędne Skały (BS), and Torfowisko pod Zieleńcem (TZ) (Fig. 2, Table S1). Bór na Czerwonem is a peat bog situated in the Orava–Nowy Targ Basin, near the Tatra Mountains. This site comprises co-occurring populations of *P. sylvestris*, *P. mugo*, and their natural hybrids. The Błędne Skały and Torfowisko pod Zieleńcem populations are located in the Stołowe and Bystrzyckie Mountains, respectively. These sites host individuals representing both taxa, as well as numerous phenotypically intermediate forms. The Błędne Skały population, estimated to be 7,000–8,000 years old, is found on the crests of Cretaceous sandstone formations [57]. Torfowisko pod Zieleńcem is the largest of the hybrid zones studied, encompassing approximately 232 hectares of peat bog habitat, divided into three subpopulations by forest patches and access routes [56, 58, 59].

### DNA extraction and SNPs genotyping

Field sampling (2021–2023) was conducted under permits issued by the Polish Ministry of Climate and Environment (DOP-WPN.61.116.2021.MGr; DOP-WOPPN.61.35.2022.WH) and the Polish State Forests (ZG.7021.2.2021). We morphologically classified individuals in the field as parental or hybrid types [56, 66]. Prof. Witold Wachowiak and Dr. Bartosz Łabiszak conducted the formal identification of the plant material. No voucher specimen was deposited as a few fresh needle samples were used only for DNA isolation. DNA was extracted using the Genomic Mini AX Plant Kit (A&A Biotechnology, Poland). DNA concentration was quantified using the Qubit 4 fluorometer with the Broad Range Assay Kit, and all samples were diluted to a working concentration of 40 ng/µl. Genotyping was performed using the Axiom_PineGAP SNP array which initially produced 49,829 SNPs, containing polymorphisms identified in transcriptome sequences and during the resequencing of candidate genes in the studied pine species [67]. We retained only high-quality markers classified as “Best and Recommended” (QC ≥ 90, DQC ≥ 0.82) in the Axiom Analysis Suite. Because LD in pines is extremely low (< 200 bp in coding regions [68–70] and rare SNPs mainly add noise without improving population structure inference, we excluded redundant SNPs in LD and those with low MAF. Therefore, further filtering excluded SNPs with a minor allele frequency (MAF) < 0.05 and those in linkage disequilibrium (LD > 0.8), resulting in 7,390 SNPs used for downstream analyses.

### Genetic assignment of samples

To assess the distribution of genetic variance and identify the broad genetic structure of the studied pines within the hybrid zones we performed principal component analysis (PCA) using the *adegenet* R package [71, 72]. Next, we used Latent Factor Models implemented in the *LEA* R package [73] to identify a number of ancestral clusters and to classify individuals from the hybrid zones based on their ancestry coefficients into predefined genotypic classes. The number of ancestral clusters (K) was tested from K = 1 to 10, with 10 replicates per value, to determine cross-entropy. The graphical illustration of individual ancestry coefficients was plotted using POPHELPER Structure Web App v1.0.10 [74]. As the optimal K was 2 corresponding with *P. sylvestris* and *P. mugo* as ancestry (see Results), we classified our sampled trees based on the estimates of the ancestry coefficient expressed as Q-scores into parental species (*Pinus sylvestris*, *Pinus mugo*) and hybrids.

Individuals with ≥ 97% ancestry from one taxon were designated as “pure species” (PS and PM). Those with 40–60% ancestry from each parent were classified as putative F1 hybrids. The remaining individuals were assigned to either *P. sylvestris* or *P. mugo* hybrids based on their predominant genomic ancestry (referred hereafter as H_PS (between 3% and 40% of *P. mugo* ancestry) and H_PM (between 3% and 40% of *P. sylvestris* ancestry), respectively). Such division, especially in case of pure individuals, is supported by our previous studies, where we have not reported significant admixture in reference stands of studied species. The spatial distribution of genetic classes was visualized using the *ggplot2* [75] and *ggmap* [76] packages in R [77], with Google Maps API tiles (zoom levels: BC = 16, BS = 17, TZ = 15). Data handling was performed using *dplyr* [78], *forcats* [79], *tidyr* [80], and *tidyverse* [81]. Figure layouts were refined using *ggpubr* [82] and *cowplot* [83].

### Outlier analysis

Outliers detection was performed using three complementary approaches. Prior to analysis, SNP data were converted using the *dartR* package [84] to generate input formats compatible with pcadapt, OutFLANK, and BayeScan. The first method, pcadapt v4.3.3, detects candidate loci under selection by assessing their association with principal components (PCs) of genetic variation, without relying on predefined population assignments [85]. We tested values of K (the number of retained PCs) from 1 to 15 and selected K = 2 for final analysis, based on scree plot inspection and explained variance. Loci were ranked by their Mahalanobis distance from the center of the PC distribution, and statistical significance was assessed via false discovery rate (FDR) correction. The second approach, OutFLANK v0.2, identifies outliers by modeling the distribution of F_ST_ values expected under neutrality. It trims the extreme tails of the empirical F_ST_ distribution to estimate the parameters of the null distribution, which is then used to identify loci with unusually high F_ST_ values indicative of divergent selection. This method is designed to be conservative, minimizing false positives, and does not require prior knowledge of population structure beyond population labels [86]. The final method, BayeScan v2.1, applies a Bayesian framework to estimate the posterior probability that each locus is under selection. It models allele frequencies using population-specific and locus-specific F_ST_ coefficients, distinguishing between selection and demographic effects. A reversible-jump MCMC algorithm is used to sample the parameter space, and loci with strong support for selection (based on posterior odds) are identified [87]. We applied a prior odds ratio of 10, following standard recommendations for moderate stringency.

We performed multiple levels of analyses, comparing different sets of individuals with two main reference groups – all allopatric populations of *P. sylvestris* and all allopatric populations of *P. mugo*. The studied groups included whole genetic classes (e.g. all individuals from three hybrid zones assigned to hybrid *P. sylvestris* class; H_PS) and individuals assigned to the genetic classes from each hybrid zones (e.g. all *P. sylvestris* hybrids from Bór na Czerwonem; BC_H_PS). Comparisons within and between reference populations were done in order to exclude genetic selection not associated with the contact zones.

To control the false discovery rate across all three methods, only SNPs with q-values < 0.1 - calculated using the *qvalue* package [88] - were retained as candidate outliers. Functional annotation of outlier loci was performed using the BLASTx algorithm [89] implemented in Diamond within the OmicsBox platform [90]. For SNPs derived from transcriptome datasets, we aligned full unigene sequences to improve annotation quality and retained only those SNPs located within the aligned regions. Functional classification included InterProScan domain analysis [91], Gene Ontology (GO) mapping [92, 93], and the merging of GO terms across annotation methods.

### Ancestry informative markers, hybrid index, and genomic clines

To complement ancestry estimates obtained from LEA, we calculated hybrid indices for all individuals sampled from the contact zones using the *gghybrid* R package [94], which applies a Bayesian genomic cline model to estimate the proportion of ancestry derived from each parental species. Pure, allopatric *P. mugo* populations were defined as parental group S0, and pure *P. sylvestris* populations as parental group S1. The model was run under default parameters with 6,000 MCMC iterations and a burn-in of 2,000 and repeated three times to ensure consistency of parameter estimates. Convergence of MCMC chains was verified using the Gelman–Rubin diagnostic (*N* = 10,000), with values < 1.1 interpreted as evidence of satisfactory convergence across independent runs.

Additionally, we identified ancestry-specific markers (AIMs) by comparing allele frequencies between allopatric reference populations of *P. sylvestris* and *P. mugo*. Markers were retained if the allele frequency was ≥ 0.97 in one species and ≤ 0.03 in the other, representing putatively fixed, diagnostic variants useful for tracing genomic ancestry and introgression. We then assessed the distribution of these AIMs in hybrid individuals to investigate potential biases in ancestry contributions. Specifically, we calculated the mean allele frequency of species-specific SNPs within hybrids to evaluate whether AIMs exhibited a consistent shift towards one parental species. This analysis was extended across multiple hybrid zones to assess spatial consistency in AIM distributions. To detect signatures of non-neutral introgression, we examined the frequency distribution of AIMs in hybrids with known hybrid indices. Deviations from expected frequencies under neutral models can signal the presence of reproductive barriers, local adaptation, or selective sweeps acting on introgressed alleles. By integrating outlier detection and allele frequency divergence, our approach aimed to identify genomic regions contributing to the maintenance of species boundaries or facilitating adaptive gene flow between *P. sylvestris* and *P. mugo* within hybrid zones.

## Results

### Genetic composition of the hybrid zones

**Fig. 3:**
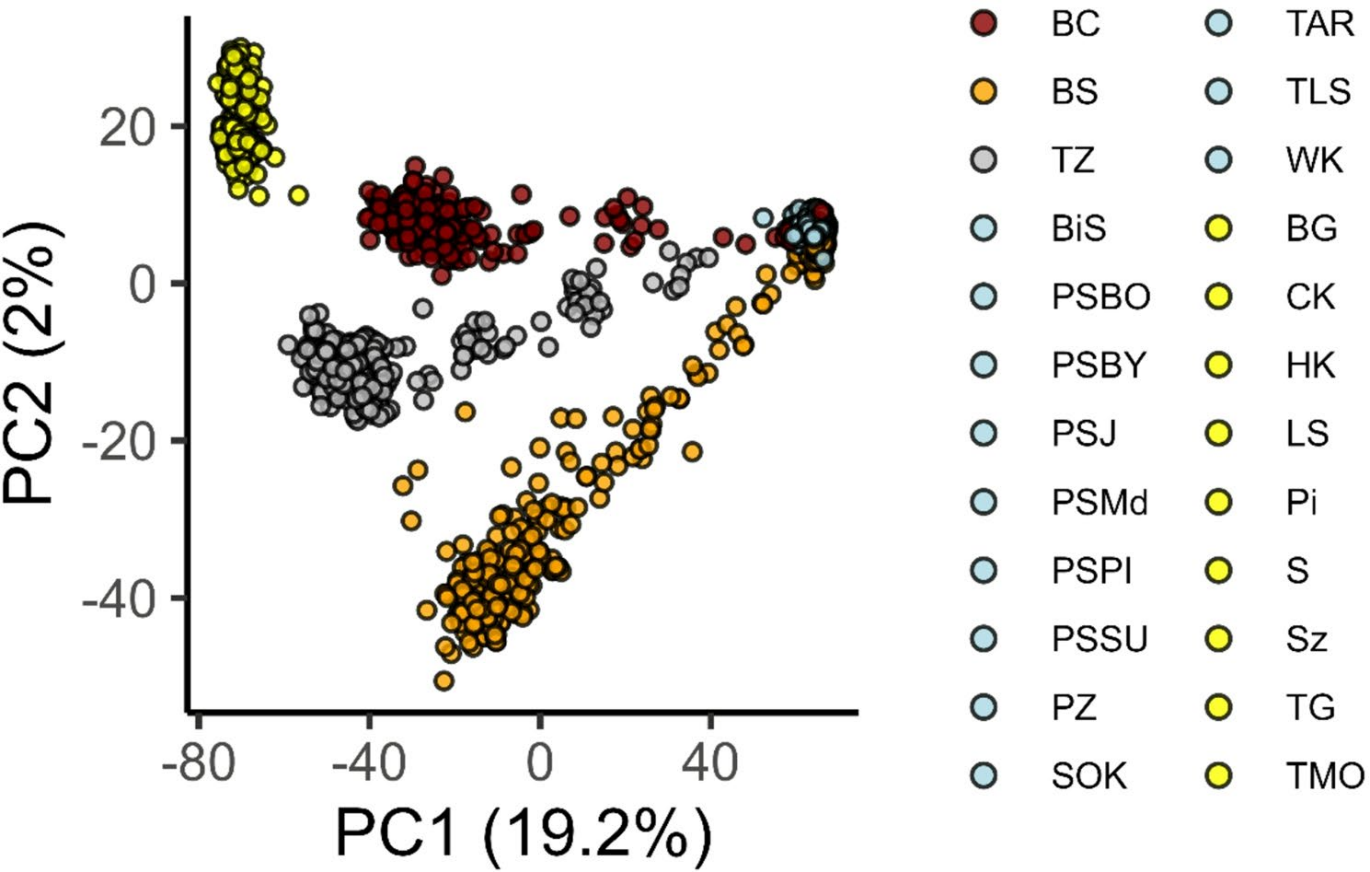
Principal component analysis (PCA) of genetic variation among sampled pine individuals based on nuclear SNP data. Colours indicate population origin: contact zones (BC, BS, TZ), reference *Pinus sylvestris* stands (shades of blue), and reference *P. mugo* populations (shades of yellow). Population codes correspond to sampling locations listed in Table S1

**Fig. 4:**
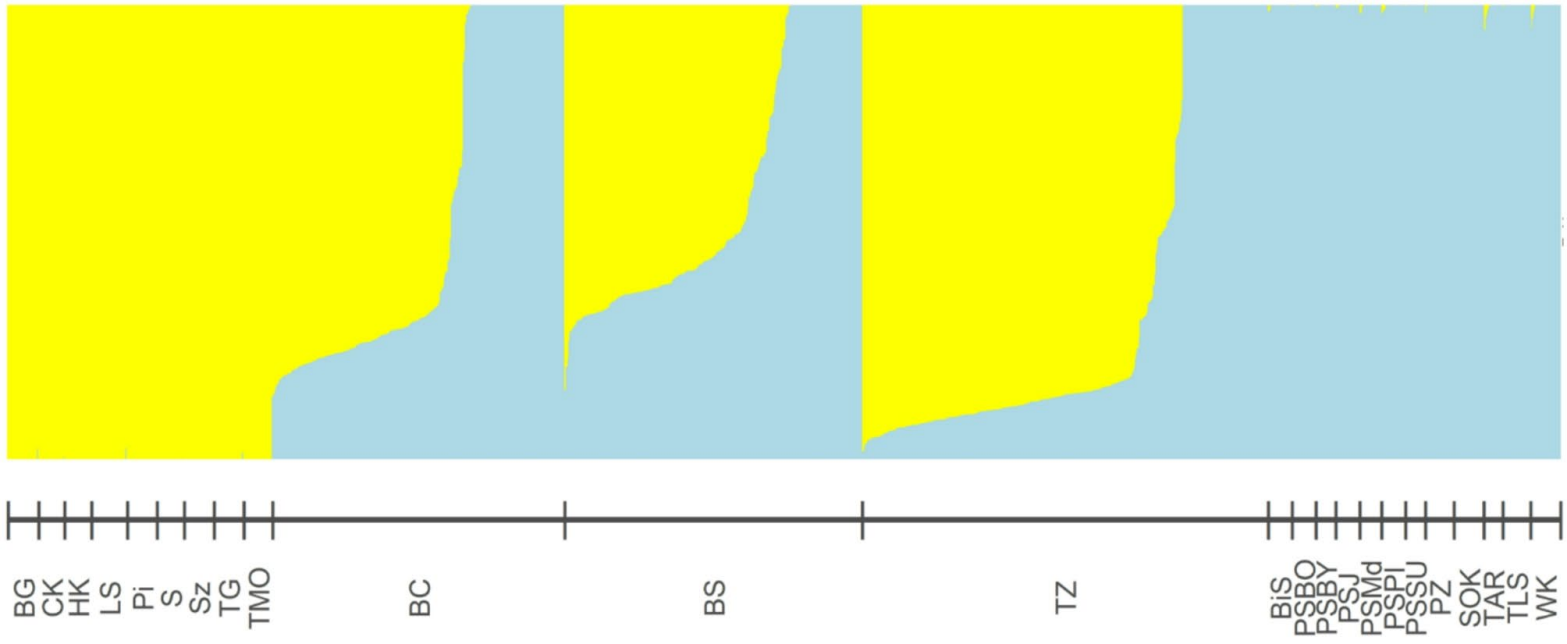
Individual ancestry proportions inferred using LEA based on nuclear SNP data (K = 2). Each vertical bar represents an individual, with assignment to one of two genetic clusters: *Pinus sylvestris* (light blue) and *P. mugo* (yellow). Individuals are grouped by population (labels below), with contact zone populations (BC, BS, TZ) positioned centrally. Populations are ordered to emphasize the transition in ancestry proportions across zones. Full population names are provided in Table S1

Principal Component Analysis (PCA) provided strong evidence that ancestry from *Pinus sylvestris* and *P. mugo* constitutes a major source of genetic variation in the studied populations. The analysis clearly separated allopatric reference populations of the two species along the first principal component (PC1), which explained 19.2% of the total genetic variance. Individuals from hybrid zones formed a genetic continuum between the parental clusters, reflecting varying degrees of admixture (Fig. 3). This was further corroborated by the species assignment analysis performed in *LEA*, which identified two main genetic groups (K = 2, Figs. 4, S1) representing the ancestry of *P. sylvestris* and *P. mugo*. We observed mixed ancestry, as evidenced by Q-scores ranging from 0.03 to 0.97 among individuals in the contact zones, while individuals from allopatric populations were classified as pure *P. mugo* or *P. sylvestris.*

Across all contact zones, genotypes with a predominance of *Pinus mugo* ancestry were most common (Fig. 2), although only three individuals were classified as pure *P. mugo* (Q-scores > 0.97), and all of these were found exclusively in the Torfowisko pod Zieleńcem (TZ) population. Individuals with 60–97% *P. mugo* ancestry predominated across all zones, with the highest number in the TZ population. The Błędne Skały population contained the highest number of putative F1 hybrids, while Bór na Czerwonem harbored the greatest proportion of pure *P. sylvestris* individuals, highlighting considerable variation in genetic composition across sites.

### Outliers detection

Outlier SNPs were detected in multiple comparisons among species and populations (Fig. S2, S3). Both OutFLANK and BayeScan identified fewer outliers compared to pcadapt, reflecting their more conservative thresholds (Fig. S4). A set of 29 SNPs was consistently identified as outliers across all three methods (Fig. S5, S5, S6). The highest number of shared outliers between any two methods (143 SNPs) was observed in comparisons between TZ F1 individuals and reference *P. sylvestris*, followed by comparisons between *P. sylvestris* hybrids (H_PS) and the same reference group (108 SNPs; Fig. S6, S7). No common outlier SNPs were found in several comparisons: F1 individuals vs. reference *P. mugo*,* P. sylvestris* hybrids from TZ vs. reference *P. sylvestris*; BC F1 individuals vs. reference *P. sylvestris*, and BC F1 individuals vs. reference *P. mugo*. The lowest number of outliers (5 common SNPs in both pcadapt and BayeScan analysis) was found between hybrid *P. mugo* vs. reference *P. mugo*.

**Fig. 5:**
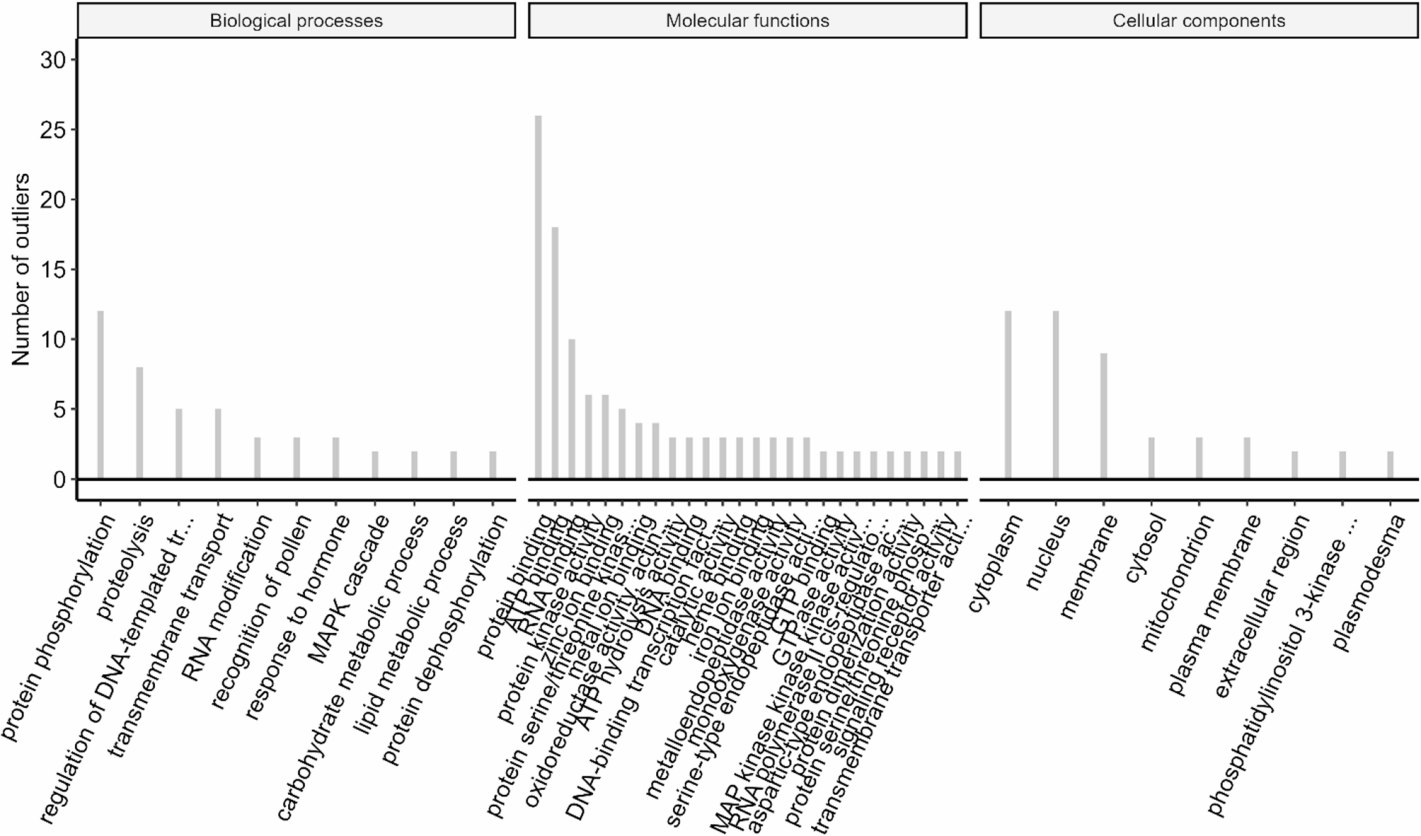
Gene Ontology (GO) term enrichment among outlier loci. Shown are GO categories associated with at least two candidate SNPs across all comparisons. Terms are grouped into three categories: Biological Processes, Molecular Functions, and Cellular Components. Bars represent the number of outlier SNPs associated with each term, highlighting functional pathways potentially involved in adaptive divergence

Out of 296 unique outlier SNPs detected in at least two methods, 288 (~ 97.3%) were successfully aligned to reference transcriptome of *P. sylvestris* [95], with 218 associated with annotated proteins based on InterPro and Gene Ontology (GO). The final set included 133 SNPs located in coding regions of known function (Fig. 5). The comparison between hybrid *P. sylvestris* and its reference populations revealed the greatest number of biologically and functionally annotated GO terms, followed by comparisons between F1 individuals from Torfowisko pod Zieleńcem and *P. sylvestris* (Fig. S8, S9, S10).

**Fig. 6:**
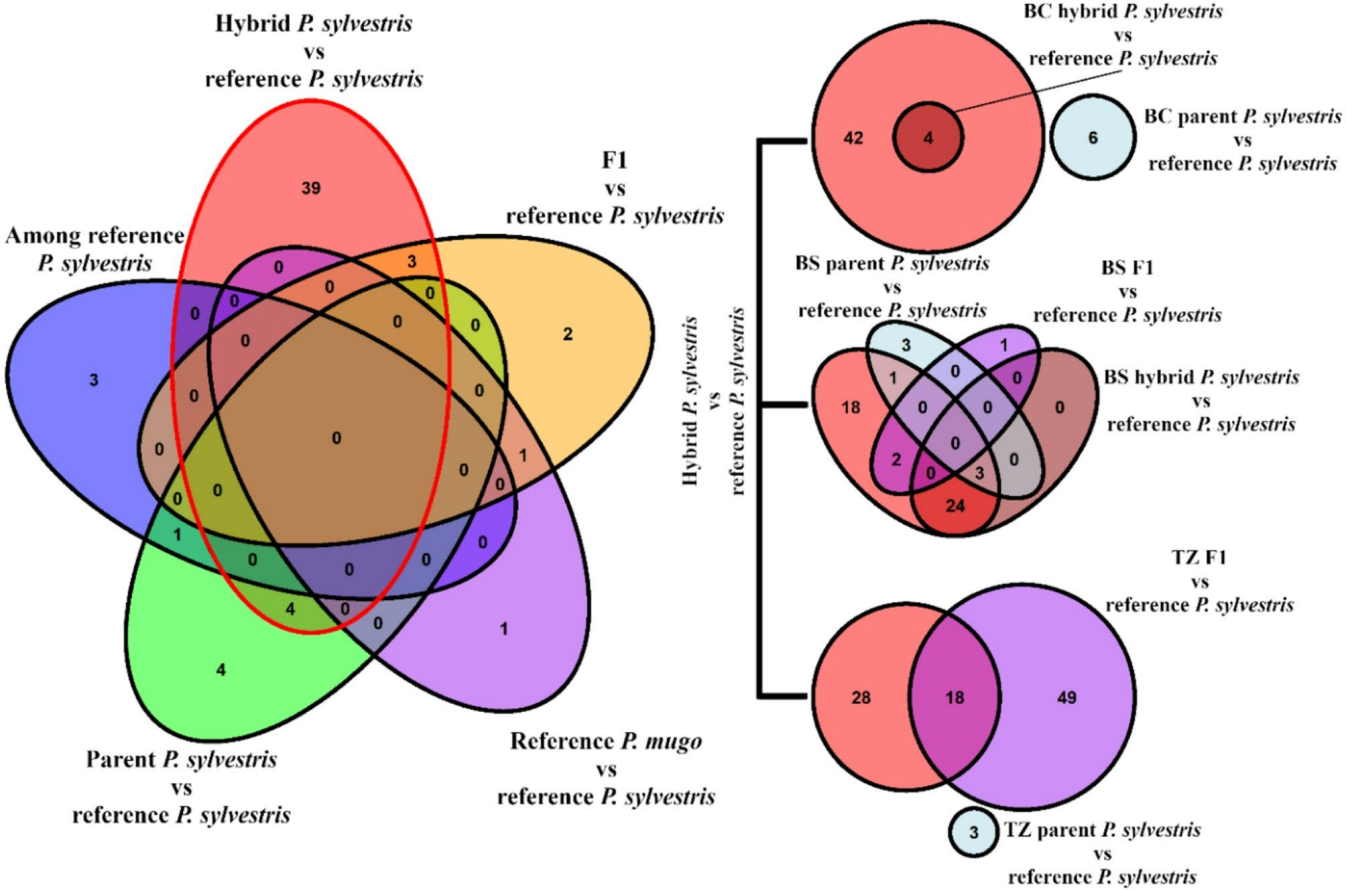
Overlap of outlier loci identified in pairwise comparisons involving hybrid classes, parental *Pinus sylvestris*, and reference populations. Left: Venn diagram summarizing shared and unique outliers detected in global comparisons. Right: Zone-specific comparisons for Bór na Czerwonem (BC), Błędne Skały (BS), and Torfowisko pod Zieleńcem (TZ) contact zones show the extent of overlap in outlier loci between hybrid, F1, and local parental classes versus reference *P. sylvestris*

Some outlier loci were common for different comparisons of reference and contact zones groups including several genes related to biological processes and signal transduction such as receptor-like protein, protein kinase, and genes related to cellular transport (Fig. 6, Table S2). All reference *P. sylvestris* had only one common outlier with the pure *P. sylvestris* species class from contact zones (methionine aminopeptidase 2B) that was also found in comparison among reference *P. mugo* populations (Table S3).

Overall, *P. sylvestris* showed a broader and more complex landscape of genomic divergence under selection compared to *P. mugo*. The number of outlier loci detected in comparisons involving *P. sylvestris* was consistently higher (Table S2) than those involving *P. mugo* (Table S3), particularly in the Błędne Skały parental *Pinus sylvestris* (BS_PS). Interestingly, in this contact zone, we detected a notably high number of unique outlier loci (*n* = 66) in the comparison of hybrid group (H_PS) to the reference *P. sylvestris* populations. In contrast, only one or no unique outliers were identified in the corresponding comparisons of hybrid groups from the Bór na Czerwonem (BC) and Torfowisko pod Zieleńcem (TZ), respectively. Unexpectedly, no outliers were shared between the TZ and BC peat bogs, despite their broadly similar habitat characteristics. The only instance of outlier sharing between H_PS was observed between BC and BS zones, where five loci overlapped (Fig. S11). Several outlier loci were consistently detected across different comparisons involving parental PS and hybrid groups. Those outliers were located in several genes of known functions including Receptor-like protein 4, Serine/threonine-protein kinases (D6PK, UCNL-like) and U-box domain-containing protein 34. Some loci were population-specific such as small GTP-binding protein and ribonucleoside-diphosphate reductase, unique to Błędne Skały PS and NPGR2-like protein was exclusive to the Torfowisko pod Zieleńcem PS group.

In contrast, introgressed *P. mugo* had fewer outliers overall, a lower number of shared outliers across hybrid and parental comparisons, and several exclusive outliers found only in reference PM populations, not observed in contact zones or hybrid groups. We found no evidence of extensive outlier sharing between the Torfowisko pod Zieleńcem (TZ) and Bór na Czerwonem (BC) populations, with only three outliers shared between them. In contrast, both populations possessed a relatively higher number of unique outliers, with eight loci detected exclusively in each. In the Błędne Skały (BS) hybrid group, only a single unique outlier was identified, indicating limited divergence at outlier loci. Several annotated outliers correspond to transcriptional regulators and metabolic transporters including bHLH transcription factor RHL1, auxin response factor 10, and serine/threonine-protein kinase VPS15 that play roles in development, signaling, and response to environmental stress. Other outliers included glutamine synthetase, adenine nucleotide transporter BT1, and molybdenum cofactor sulfurase imply metabolic differentiation, especially in nitrogen and energy pathways. A small number of loci were shared between hybrids and interspecific comparisons, such as the thyroid adenoma-associated protein homolog, identified in both F1 vs. PS and PM vs. PS comparisons.

### Ancestry informative markers, hybrid index and genomic clines

**Fig. 7:**
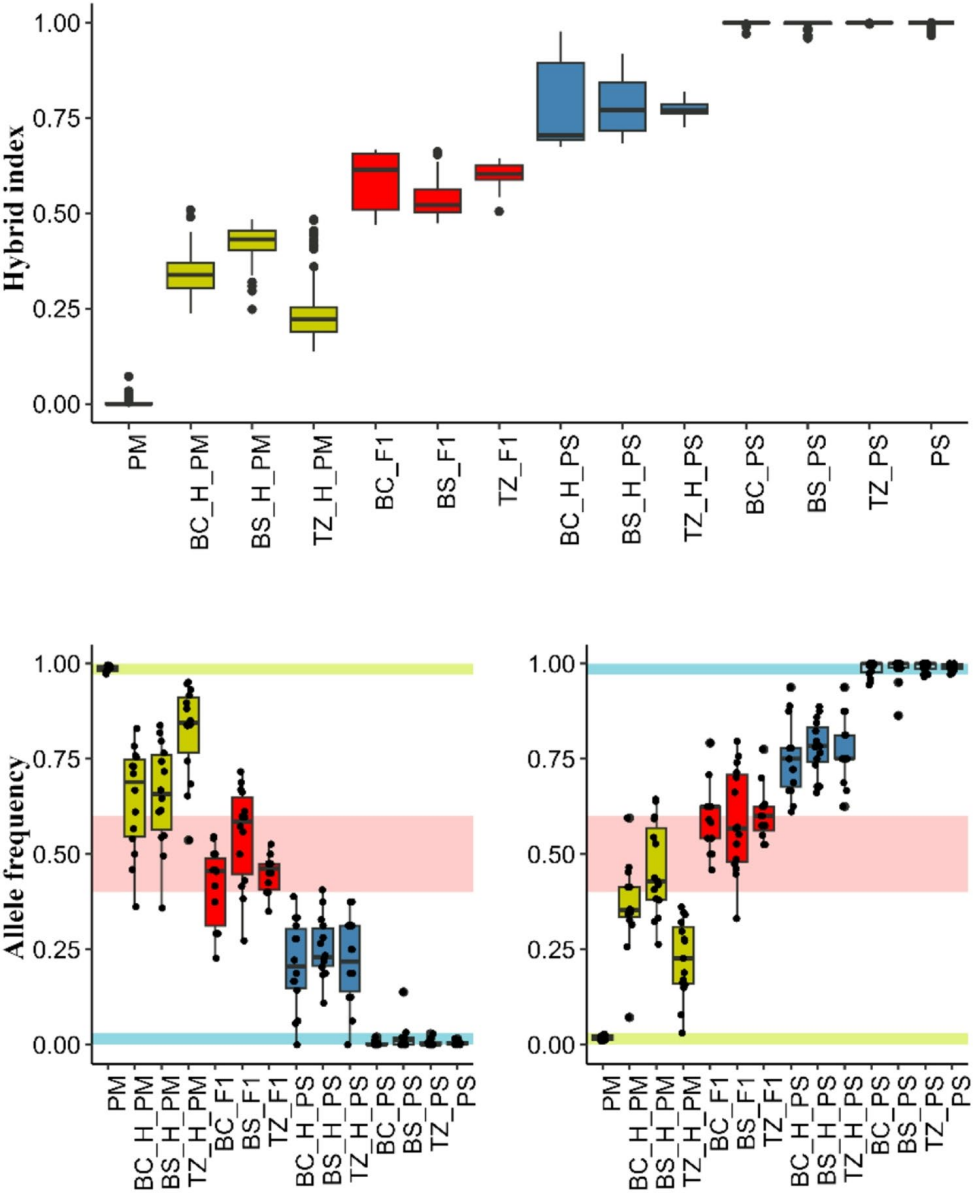
Hybrid Index calculated for all the studied sympatric populations (top) and frequencies of Ancestry Informative Markers (AIMs) in reference and hybrid populations. Description of populations as in Table S1. Colours correspond to pure *P. mugo* (yellow), hybrid PM (dark yellow), F1 (red), hybrid PS (dark blue) and pure *P. sylvestris* (light blue)

Hybrid index analysis confirmed the presence of highly admixed individuals in all contact zones, as initially detected by PCA and STRUCTURE-like clustering. (Fig. 7). Ancestry assignments were highly consistent across approaches, with a strong correlation between *LEA* admixture coefficients and hybrid index estimates (*r* = 0.99; Fig. S12), indicating the robustness of both methods for detecting hybrid ancestry. Additionally, hybrid index analysis reveals asymmetric introgression across zones. The Torfowisko pod Zieleńcem (TZ) contact zone shows the strongest shift toward *P. mugo* ancestry, while Błędne Skały (BS) also shifts in the same direction but less strongly. In contrast, the Bór na Czerwonem (BC) zone exhibits a more balanced admixture, with some hybrid groups (e.g., BC_H_PS) skewed slightly toward *P. sylvestris*. These differences highlight spatial variation in gene flow dynamics, with asymmetry toward *P. mugo* more pronounced in TZ, suggesting localized ecological or demographic influences [96–98].

We identified 29 ancestry-specific outlier loci, 15 fixed or nearly fixed in *Pinus sylvestris* and 14 in *P. mugo* reference populations (Fig. 7; Table S4). 10 of those loci also overlapped with those identified as selection outliers, suggesting that a subset of ancestry-informative markers (AIMs) may be under directional selection. Notably, only three of these overlapping loci were successfully annotated: abscisic acid receptor PYL4, bHLH transcription factor RHL1, and thyroid adenoma-associated protein homolog [99] – genes with putative roles in stress response, development, and reproductive regulation. Surprisingly, high frequencies of *P. sylvestris*-fixed alleles were observed across numerous loci in *P. mugo*-like hybrids (H_PM) from all contact zones, with this pattern being most pronounced in individuals from the Torfowisko pod Zieleńcem (TZ) population. Despite an overall genomic composition skewed toward *P. mugo*, hybrids in the TZ_H_PM group consistently exhibited elevated frequencies of PS-specific alleles. At several loci allele frequencies was near 0.5, indicating substantial and potentially non-neutral introgression into a *P. mugo*-dominated background. Among these, only locus 117434739 was associated with a gene of known function – vacuolar protein sorting-associated protein 29, which may play a role in protein trafficking and cellular homeostasis.

In contrast, introgression of *P. mugo*-fixed alleles into *P. sylvestris*-like hybrids (H_PS) was limited. Several loci consistently showed low frequencies of PM alleles across H_PM groups, particularly in the BS and BC contact zones, often not exceeding 0.25. Only in the TZ_H_PM group did these loci display elevated frequencies, broadly consistent with their overall hybrid index. Among these outliers, some were located in genes with known functional roles, including bHLH transcription factor RHL1 (117443799) and putative pumilio homolog 8, chloroplastic (117420230), both implicated in transcriptional regulation and post-transcriptional gene silencing.

## Discussion

### Hybridization and population structure

Our results demonstrate that natural hybridization between *Pinus sylvestris* and members of the *Pinus mugo* complex is widespread in the studied contact zones. All three sympatric populations exhibited a continuous spectrum of phenotypic forms, ranging from typical *P. sylvestris*, through hybrids representing a wide array of transgressive phenotypes, to typical shrub-like *P. mugo*. The most prevalent genetic class across all sympatric stands were hybrids, the majority of which showed predominant *P. mugo* ancestry. This asymmetry was also recorded in Slovakian hybrid zones of the species, using microsatellite and iPBS nuclear markers [97, 98], what shows a consistency in the hybridization scheme within this system, apart from marker type. However, the genetic composition of individuals across the hybrid zones confirmed the presence of a full spectrum of genomes spanning from pure *P. sylvestris* to nearly pure *P. mugo*, with a continuum reflected in the smooth distribution of hybrid indices (i.e., proportions of *P. sylvestris* vs. *P. mugo* ancestry).

Yet, despite the clear morphological resemblance of some individuals to *P. mugo*, the genomic ancestry spectrum did not fully mirror the phenotypic variation. We observed mismatches between genotype-based ancestry and phenotype, including phenotypically intermediate but genetically pure individuals, as well as genetically admixed individuals with pure-like morphology. Such patterns may result from phenotypic plasticity, environmental effects, or the polygenic basis of adaptive traits, and have also been reported in previous studies [100–102]. A more detailed analysis is provided elsewhere [103].

A large number of individuals in the hybrid zones were genetically almost pure *P. sylvestris* as demonstrated also in PCA analysis, whereas virtually none were pure *P. mugo* (only three individuals across all sites exhibited >97% *P. mugo* ancestry). Although some possible F1 individuals were found, most of the trees resulted from backcrossing with one of the parental species or hybrids of different ancestry proportions. Moreover, trees classified as *P. mugo* hybrids from all hybrid zones always had less than 90% of the PM ancestry. Notably, we found no evidence of introgression in the genomes of parental taxa outside the hybrid zones. This result confirms earlier SSR and SNP evidence that interspecific gene flow is confined to pine contact zones, with no admixture in allopatric parental populations [54, 103]. As a more detailed analysis of hybrid zone formation and evolutionary dynamics in this system is presented elsewhere [103], we focus here on the molecular signatures of introgression and selection in hybrids.

### Patterns of introgression

The genomic data from thousands of SNPs allowed us to characterize the patterns of introgression across the genome in these hybrid zones. We found that hybridization between *P. sylvestris* and *P. mugo* is highly asymmetrical, with a strong bias in hybrids with the majority of *P. mugo* ancestry. As assumed that most loci were subjected to introgression and can be freely exchanged between the hybridizing species – meaning that the probability of having a variant derived from a putative parent must be proportional to the hybrid index. Interestingly, we found also that some loci were introgressed more frequently than expected considering only the hybrid index of an individual. For example, we observed that a *P. sylvestris*-derived allele at a glutamine synthetase gene (GS1b), which is normally rare in allopatric *P. mugo* (frequency < 10%), occurred at a much higher frequency in the *P. mugo*-like hybrids (up to ~ 80% in one zone). Glutamine synthetase is involved in nitrogen metabolism and has been linked to plant water-stress responses [104, 105]. The introgression and increase of the *P. sylvestris* variant in *P. mugo* hybrids suggests adaptive value – potentially helping them cope with the waterlogged, nutrient-poor peat bog conditions, especially because this mutation is nonsynonymous. Similarly, a MED15a gene allele characteristic of *P. sylvestris* (and nearly fixed in allopatric *P. sylvestris*) was found to have introgressed into hybrid *P. mugo* individuals that showed a notable increase in the frequency of the *P. sylvestris* variant at this locus. MED15a encodes a transcriptional regulator of stress-response genes (including heat-shock proteins) and was previously noted to differ between northern and southern *P. sylvestris* populations [106–109]. Introgression of the *P. sylvestris* allele into *P. mugo* in the hybrid zones (it was absent in pure *P. mugo* populations) again points to potential selective benefits conferred by *P. sylvestris* alleles. Another nonsynonymous mutation was in geraniol 8-hydroxylase, which is an enzyme that catalyses reaction producing 8-Hydroxygeraniol, which is described as a potential insect repellent [110]. The variant characteristic within the reference PM populations was introgressed into BS PS hybrids and TZ F1s. These cases exemplify adaptive introgression, where gene flow introduces alleles that improve fitness in the recipient species’ environment.

Indeed, we found also examples of alleles that remain species-specific and resist introgression despite extensive hybridization towards *P. mugo*. One striking example is a nonsynonymous mutation in a basic helix-loop-helix (bHLH) transcription factor gene (putatively *RHL1*), which is fixed for alternate alleles in the parental species (one allele fixed in *P. mugo*, the other fixed in *P. sylvestris*). In the hybrid populations, the *P. mugo*-derived allele at this locus remained nearly fixed among *P. mugo* hybrids, effectively acting as a barrier locus that maintains species differentiation. RHL1 is known to regulate root hair development in grasses [111–113], a trait potentially crucial for nutrient uptake in bog soils. The maintenance of the *P. mugo* allele exclusively in hybrids suggests that the *P. sylvestris* variant of this gene has likely a negative impact on fitness in the peat bog environment or in the genomic background of *P. mugo*. In general, such loci that remain unintrogressed (or show deficient introgression of the alternate allele) likely reflect selective constraints – they may underlie key species-specific adaptations or genetic incompatibilities. This pattern aligns with theoretical expectations of introgressive hybridization under selection, where neutral or advantageous alleles move freely between species, while maladaptive or incompatible alleles are purged from the opposite background [114, 115]. It also aligns with observations in other tree species hybrid zones (e.g., spruces and oaks), where asymmetric introgression and locus-specific barriers produce a patchwork genome structure [20, 29].

### Asymmetric selection

Despite the asymmetrical gene flow and high bias favoring the repeated formation of *P. mugo*-like hybrids across hybrid zones, we found that natural selection imposes substantially stronger evolutionary constraints on *P. sylvestris* (and hybrids with a large *P. sylvestris* genomic component) compared to *P. mugo*. The results of genome scan analyses for loci under selection fully support this conclusion: we detected a much higher number of outlier SNPs in comparisons involving *P. sylvestris* genomes than in those involving *P. mugo* genomes. In particular, hybrid individuals genetically classified as *P. sylvestris* hybrids (i.e. those with majority of *P. sylvestris* ancestry) showed a large set of allele frequency outliers when compared to allopatric *P. sylvestris* reference populations. Likewise, even “pure” *P. sylvestris* individuals growing inside the hybrid zones – which had no detectable *P. mugo* introgression as confirmed by their nuclear ancestry, morphology, and a diagnostic *cpDNA* marker (trnL-trnF locus with different variants for *P. mugo* complex and *P. sylvestris*), strictly proving their *P. sylvestris* paternal origin [54, 103, 116, 117] – exhibited numerous outlier loci distinguishing them from reference allopatric populations. By contrast, *P. mugo*-like hybrids (with majority *P. mugo* ancestry) yielded far fewer outlier loci in analogous comparisons to allopatric *P. mugo* references (Fig. S13). However, this results can be also slightly impacted by the differences between chosen reference populations – *P. mugo* populations were divided into two clusters (Sudeten vs. Carpathian populations), whereas *P. sylvestris* formed one cluster (Fig. 3). These patterns of a much broader and more complex landscape of genomic divergence under selection in *P. sylvestris* indicate that in the unfavorable conditions of peat bogs, individuals of *P. sylvestris* ancestry undergo stronger selection. It seems that their genomic background is less fit compared to that of *P. mugo*, which results in a lower number of hybrid individuals with dominant PS ancestry found in all hybrid zones. Peat bog environments lie at the edge of the ecological niche for *P. sylvestris* – a widespread generalist typically found on more mineral-rich soils at lower elevations [118, 119]. This aligns with our field observations within hybrid zones, where *P. sylvestris* trees were typically restricted to the drier margins of peat bogs and largely absent from the wettest, central areas. The strong selection signatures detected in *P. sylvestris* from these zones suggest that only genotypes with specific adaptive traits can establish and persist under such marginal conditions. These may include individuals carrying pre-adapted alleles from standing genetic variation within *P. sylvestris*, or hybrids inheriting adaptively introgressed alleles. Importantly, our data indicate that adaptation of *P. sylvestris* to the bog environment does not primarily rely on introgression from *P. mugo*. If survival were largely mediated through adaptive introgression, we would expect hybrids and backcrossed individuals to dominate, with pure *P. sylvestris* individuals being rare or absent. However, we identified several genetically pure (or nearly pure) *P. sylvestris* pines thriving within the contact zones. These individuals showed signals of divergent selection on their own genomic backgrounds – relative to allopatric *P. sylvestris* – rather than extensive *P. mugo* introgression. Among these pure individuals, we detected outlier SNPs in coding regions of genes such as MetAP2B (methionine aminopeptidase 2B), FAAH (fatty acid amide hydrolase isoform X2), and OMA1 (mitochondrial metalloendopeptidase). These genes play key roles in abiotic stress response, energy regulation, and cellular homeostasis, suggesting that selection in peat bogs may act on standing genetic variation to promote physiological resilience in *P. sylvestris* [120–127].

However, hybrids with predominantly *P. sylvestris* ancestry showed some molecular signatures of adaptive introgression. These hybrids were rare across hybrid zones and far outnumbered by *P. mugo*-like individuals, suggesting strong selection against them. Notably, in these *P. sylvestris*-like hybrids, we observed fixed *P. mugo*-derived allelic variants at several loci, indicating that introgressed alleles were facilitating their persistence and may act as a genomic rescue mechanism in an otherwise less adapted genetic background. Several of these introgressed variants occurred in genes involved in environmental response and stress regulation, including RLP4 (cell wall integrity and directional growth), Expansin B1 (morphological plasticity under abiotic stress), and UCNL-like kinase (signal transduction). One of the most ecologically informative outliers and a nonsynonymous mutation was dehydrin 9, a gene associated with cold and dehydration tolerance and previously identified as a candidate for climatic adaptation in conifers [68]. In *P. sylvestris*-like hybrids, the *P. mugo*-derived allele at this locus was often fixed or present in the heterozygous state. This strongly supports the hypothesis that adaptive introgression at stress-relevant loci contributes to the persistence of *P. sylvestris* genetic backgrounds in these hybrid zones under the adverse environmental conditions and nutrient limitations characteristic of peat bog ecosystems [128–131].

The genomic background of *P. mugo* appears more stable and is subjected to weaker selective filtering in such environments. This likely reflects *P. mugo*’s ecological specialization for harsh, nutrient-poor, and waterlogged conditions typical of the mountain and subalpine habitats [132]. For example, *P. mugo* is known for its higher tolerance to abiotic stressors such as low temperatures, poor soil aeration, and high water table levels – traits that are beneficial in peat bog ecosystems [133]. These adaptive traits appear to provide a consistent fitness advantage and are likely retained in *P. mugo* hybrids despite gene flow from *P. sylvestris*, helping maintain their ecological performance under local conditions. The historical context of these stands further supports such observations. Generally, *P. mugo* ancestry and its *cpDNA* variants dominates in all three studied contact zones and most likely inhabited those areas before *P. sylvestris*. During the postglacial period, *P. sylvestris* migrated northward from southern refugia into northern Europe. This migration led *P. sylvestris* to advance into post-glacial peat bogs already occupied by established *P. mugo* populations in these habitats [103, 134]. Consequently, *P. mugo* underwent more generations of selection, enhancing its ecological advantage and optimizing its fitness in situ.

Several outliers found in a group of samples with a predominance of *P. mugo* ancestry included bHLH transcription factor RHL1, mediator of RNA polymerase II (Med15a), and serine/threonine-protein kinase VPS15. We also found a few SNP that showed selection patterns between reference *P. mugo* stands that were derived from genes including subtilisin-like proteases (SBT2.2, SBT2.5), and argonaute 1 (AGO1).

Taken together these findings indicate, that *P. sylvestris* persists in these peat bog environments either as a pure taxon shaped by strong selection on standing genetic variation or as a hybrid lineage rescued by targeted introgression. In contrast, individuals of predominant *P. mugo* ancestry maintain high fitness across zones, likely due to their preadaptation of their ancestors to such harsh ecological conditions. Similar asymmetrical selection dynamics have been reported in other hybrid zones where one species is locally adapted and the other is a more generalist or a recent colonizer. For example, in *Populus* hybrid zones, native species often exhibit genomic stability, while introgressed genotypes from less-adapted species are selectively filtered [135]. In *Helianthus*, hybridization has facilitated ecological expansion only when adaptive traits were acquired from the locally adapted parent [100, 101]. Likewise, in hybrid zones of *Quercus* and *Pinus*, species pre-adapted to local environmental conditions have been shown to impose strong selective pressures on their hybridizing counterparts, resulting in directional or asymmetrical introgression [40, 136, 137]. These cases, like our findings, highlight that even within a shared physical habitat, hybridizing species may experience very different selective regimes depending on their ecological proximity to the local adaptive optimum.

### Convergence evolutionary outcomes

Our comparative analysis of outlier loci across the zones reveals a notable degree of overlap in genetic targets of selection. A majority of outlier SNPs identified in one hybrid population were also detected as outliers in at least one other population, and several loci showed consistent signals of selection across all three zones. For example, a SNP in the RLP4 gene, encoding a receptor-like protein kinase associated with cell wall integrity and directional growth in *Arabidopsis*, was identified as an outlier in hybrids or local *P. sylvestris* across all sites. The recurrent selection on this gene suggests that modulation of cell wall properties may represent a common adaptive requirement in pine hybrids – potentially to withstand mechanical stress from snow, wind, or water saturation in open bog environments. Other genes including serine/threonine-protein kinases (D6PK, UCNL-like) and U-box domain-containing protein 34 were frequently detected across *P. sylvestris* and hybrids, suggesting roles in cell signalling, stress responses, and/or reproductive compatibility [138, 139]. An outlier SNP in Expansin B1, a gene involved in cell wall loosening and root growth, was detected in multiple zones. Expansins have been linked to enhanced root development and abiotic stress tolerance in various plant species [129–131], suggesting that the selection on this gene may promote rooting in loose, acidic peat substrates or enhance physiological resilience under environmental stress [100]. 

Contrary to our initial expectation, we did not observe strong genetic distinctiveness in the Błędne Skały (BS) hybrid zone relative to the Torfowisko pod Zieleńcem (TZ) and Bór na Czerwonem (BC) zones. BS is the only site where hybrids occupy sandstone rock formations with a shallow soil layer, and it also shows a subtle shift toward *P. sylvestris* ancestry, pointing to a potentially stronger role of environmental selection in shaping hybrid composition. Although there was only one outlier purely unique for PS group from BS hybrid zone (putative clathrin assembly protein At2g25430, associated with salt stress [140, 141]), we detected many outlier loci present only in grouped PS hybrids and the PS hybrids from BS zone, such as described before expansin B1 and dehydrin 9, but also pfkB-like carbohydrate kinase family protein, associated with controlling of plant development [142]. However, the fact that most outlier loci were shared among multiple sites suggests that convergent selection pressures outweigh divergent, site-specific responses, reinforcing the idea of parallel evolution in these independently formed hybrid zones. This suggests that adaptation in contact zones populations in PS occurs through selection within the species with support of adaptive introgression from PM in some cases.

## Conclusions

Our study demonstrates consistent and repeatable genomic patterns of hybridization between *Pinus sylvestris* and *P. mugo* across three distinct contact zones, despite differences in local climate and biogeographic history. All zones were dominated by hybrid individuals, with asymmetric introgression favoring *P. mugo* ancestry and stronger selection signatures observed in *P. sylvestris*. However, contrary to expectations of parallel adaptation in similar habitats, we detected only a limited number of shared outlier loci among zones, indicating that similar environments did not consistently select for the same genomic regions. Instead, local adaptation appears to involve distinct sets of loci in each hybrid zone, shaped by context-dependent selection and the unique genetic backgrounds of local populations. Certain genes, including RLP4, GS1b, expansin B1, and dehydrin 9, showed evidence of non-neutral introgression and may contribute to environmental stress tolerance in hybrids. In contrast, the genomic background of *P. mugo* remained comparatively stable, suggesting preadaptation to the ecological conditions of the contact zones. These results indicate that while the demographic and directional aspects of hybridization are repeatable, the genetic basis of adaptation in hybrid zones is largely zone-specific. This underscores the role of hybrid zones as dynamic systems where introgression and selection interact to generate locally unique evolutionary trajectories, rather than uniform responses across replicated environments.

## Supplementary Information

Supplementary Material 1.

## Data Availability

The datasets generated and/or analysed during the current study are freely available in the open access Figshare repository: DOI: 10.6084/m9.figshare.29097569.
