# Correction: Molecular signatures of adaptive introgression and selection in contact zones of closely related pine species (*Pinus* genus)

**DOI:** 10.1186/s12870-025-07737-7

**Published:** 2025-11-18

**Authors:** Sebastian Szczepański, Bartosz Łabiszak, Witold Wachowiak

**Affiliations:** 1 Department of Plant Ecology and Environmental Protection, Institute of Environmental Biology, Adam Mickiewicz University in Poznań, Uniwersytetu, Poznańskiego 6, Poznań, 61-614 Poland; 2 Department of Genetics and Environmental Interactions, Institute of Dendrology, Polish Academy of Sciences, Parkowa 5, Kórnik, 62-035 Poland

**Correction: BMC Plant Biol 25, 1423 (2025)**

**https://doi.org/10.1186/s12870-025-07490-x**

Following publication of the original article [1], the authors found that the given names and family names of the authors were mistakenly switched. The details are given below:

**Correct author names**

Given name: Sebastian

Family name: Szczepański

Given name: Bartosz

Family name: Łabiszak

Given name: Witold

Family name: Wachowiak

The corrections do not affect the conclusion of the article. The original article [1] has been corrected.
