# Supplementary material for: Molecular signatures of adaptive introgression and selection in contact zones of closely related pine species (Pinus genus)
Source: BMC Plant Biol. 2025 Oct 21;25:1423. doi: 10.1186/s12870-025-07490-x (PMC12542055; doi:10.1186/s12870-025-07490-x)

**Table S1.** Localization of four contact zones of studied pines and reference allopatric populations of parental taxa.

|  | **Acronym** | **N** | **Population/group** | **Longitude (E)** | **Latitude (N)** |
| --- | --- | --- | --- | --- | --- |
| Bór na Czerwonem  contact zone | BC_H_PM | 174 | Poland, Bór na Czerwonem; hybrids *P. mugo* | 20°2'23.64" | 49°27'37.44" |
|  | BC_F1 | 12 | Poland, Bór na Czerwonem; F1 | 20°2'23.64" | 49°27'37.44" |
|  | BC_H_PS | 9 | Poland, Bór na Czerwonem; hybrids *P. sylvestris* | 20°2'23.64" | 49°27'37.44" |
|  | BC_PS | 77 | Poland, Bór na Czerwonem; *P. sylvestris* | 20°2'23.64" | 49°27'37.44" |
| Błędne Skały  contact zone | BS_H_PM | 113 | Poland, Błędne Skały; hybrids *P. mugo* | 16°17'15.72" | 50°28'49.44" |
|  | BS_F1 | 77 | Poland, Błędne Skały; F1 | 16°17'15.72" | 50°28'49.44" |
|  | BS_H_PS | 32 | Poland, Błędne Skały; hybrids *P. sylvestris* | 16°17'15.72" | 50°28'49.44" |
|  | BS_PS | 30 | Poland, Błędne Skały; *P. sylvestris* | 16°17'15.72" | 50°28'49.44" |
| Torfowisko pod Zieleńcem  contact zone | TZ_PM | 3 | Poland, Torfowisko pod Zieleńcem; *P. mugo* | 16°24'45" | 50°20'50.28" |
|  | TZ_H_PM | 291 | Poland, Torfowisko pod Zieleńcem; hybrids *P. mugo* | 16°24'45" | 50°20'50.28" |
|  | TZ_F1 | 20 | Poland, Torfowisko pod Zieleńcem; F1 | 16°24'45" | 50°20'50.28" |
|  | TZ_H_PS | 8 | Poland, Torfowisko pod Zieleńcem; hybrids *P. sylvestris* | 16°24'45" | 50°20'50.28" |
|  | TZ_PS | 86 | Poland, Torfowisko pod Zieleńcem; *P. sylvestris* | 16°24'45" | 50°20'50.28" |
| Allopatric reference *P. mugo* | BG | 31 | Poland, Babia Góra | 19°31'50.88" | 49°34'23.52" |
|  | CK | 26 | Poland, Karkonosze Mountains, Czarny Kocioł | 15°35'5.64" | 50°47'18.6" |
|  | HK | 27 | Poland, Tatra Mountains, Hala Kondratowa | 19°56'48.48" | 49°14'51" |
|  | LS | 36 | Poland, Karkonosze Mountains, Łabski Szczyt | 15°31'53.04" | 50°47'26.52" |
|  | PI | 30 | Poland, Pilsko | 19°19'1.56" | 49°31'44.4" |
|  | S | 27 | Poland, Karkonosze Mountains, Śnieżka | 15°44'24.72" | 50°44'11.76" |
|  | SZ | 30 | Poland, Karkonosze Mountains, Szrenica | 15°30'54.36" | 50°47'33" |
|  | TG | 30 | Poland, Tatra Mountains, Grześ | 19°45'59.76" | 49°14'12.12" |
|  | TMO | 29 | Poland, Tatra Mountains, Morskie Oko | 20°4'4.8" | 49°12'0.72" |
| Allopatric reference *P. sylvestris* | PZ | 29 | Poland, Polanica-Zdrój | 16°29'51.72" | 50°23'52.08" |
|  | BIS | 24 | Poland, Stołowe Mountains, Białe Skały | 16°21'38.96" | 50°27'32.3" |
|  | SOK | 30 | Poland, Pieniny Mountains, Sokolica | 20°26'29.04" | 49°25'6.24" |
|  | TAR | 19 | Poland, Tarnawa | 22°49'46.92" | 49°6'45.72" |
|  | TLS | 28 | Poland, Tatra Mountains, Łysa Skałka | 20°6'48.96" | 49°15'53.64" |
|  | WK | 30 | Poland, Tatra Mountains, Koryciska Wielkie | 19°48'30.24" | 49°16'10.56" |
|  | PSBY | 20 | Poland, Bytów | 17°29'55.68" | 54°10'39" |
|  | PSBO | 24 | Poland, Bolewice | 16°7'4.08" | 52°23'50.28" |
|  | PSMD | 22 | Poland, Międzylesie | 16°39'17.64" | 50°8'51.72" |
|  | PSJ | 24 | Poland, Janów Lubelski | 22°24'42.12" | 50°42'0" |
|  | PSPI | 24 | Poland, Pisz-Dziadki | 21°37'21.36" | 53°30'11.52" |
|  | PSSU | 20 | Poland, Supraśl | 23°20'6.72" | 53°12'26.64" |

N – number of individuals sampled

**Table S2.** Outliers scored for listed groups when compared to Scots pine reference populations

|  |  | Grouped comparisons | | | | | Individual hybrid zone comparisons | | | | | | |
| --- | --- | --- | --- | --- | --- | --- | --- | --- | --- | --- | --- | --- | --- |
| SNP ID | Description | Among reference PS | Hybrid PS | Parent PS | Reference PM | F1 | BC hybrid PS | BS hybrid PS | BC parent PS | BS parent PS | TZ parent PS | BS F1 | TZ F1 |
| 117408287 | receptor-like protein 4 |  | **X** | **X** |  |  | **X** | **X** |  | **X** |  |  |  |
| 117426549 | serine/threonine-protein kinase UCNL-like |  | **X** | **X** |  |  | **X** | **X** |  | **X** |  |  |  |
| 117422981 | serine/threonine-protein kinase D6PK |  | **X** | **X** |  |  |  | **X** |  | **X** |  |  |  |
| 117442017 | probable carboxylesterase 17 |  | **X** |  |  |  | **X** | **X** |  |  |  |  | **X** |
| 117399376 | expansin B1 |  | **X** |  |  |  |  | **X** |  |  |  |  | **X** |
| 117404532 | SWI/SNF complex subunit SWI3A isoform X2 |  | **X** |  |  |  |  | **X** |  |  |  |  | **X** |
| 117407503 | probable chalcone--flavonone isomerase 3 |  | **X** |  |  |  |  | **X** |  |  |  |  | **X** |
| 117412986 | geraniol 8-hydroxylase |  | **X** |  |  |  |  | **X** |  |  |  |  | **X** |
| 117414888 | ATP-dependent zinc metalloprotease FTSH 7, chloroplastic |  | **X** |  |  |  |  | **X** |  |  |  |  | **X** |
| 117418131 | probable BOI-related E3 ubiquitin-protein ligase 3 |  | **X** |  |  |  |  | **X** |  |  |  |  | **X** |
| 117423292 | cytochrome P450 71AU50-like |  | **X** |  |  |  | **X** |  |  |  |  |  | **X** |
| 117427863 | hypothetical protein Mapa_017458 |  | **X** |  |  |  |  | **X** |  |  |  |  | **X** |
| 117430188 | heavy metal-associated isoprenylated plant protein 39 |  | **X** |  |  |  |  | **X** |  |  |  |  | **X** |
| 117431263 | Methionine aminopeptidase 2B | **X** |  | **X** |  |  |  |  |  |  | **X** |  |  |
| 117434579 | THO complex subunit 1 |  | **X** |  |  |  |  | **X** |  |  |  |  | **X** |
| 117434919 | AT-hook motif nuclear-localized protein 10 |  | **X** |  |  | **X** |  | **X** |  |  |  |  |  |
| 117436073 | aspartyl protease family protein 2-like |  | **X** |  |  |  |  | **X** |  |  |  |  | **X** |
| 117441547 | protein JOKA2 |  | **X** |  |  |  |  | **X** |  |  |  |  | **X** |
| 117443031 | probable LRR receptor-like serine/threonine-protein kinase IRK |  | **X** |  |  | **X** |  |  |  |  |  | **X** |  |
| 117444983 | ABC transporter B family member 15-like |  | **X** |  |  | **X** |  |  |  |  |  | **X** |  |

| 117396032 | dehydrin 9 |  | **X** |  |  |  |  | **X** |  |  |  |  |  |
| --- | --- | --- | --- | --- | --- | --- | --- | --- | --- | --- | --- | --- | --- |
| 117399143 | protein NUCLEAR FUSION DEFECTIVE 4 |  |  |  |  |  |  | **X** |  |  |  |  | **X** |
| 117400305 | vacuolar-sorting receptor 6 |  | **X** |  |  |  |  | **X** |  |  |  |  |  |
| 117406566 | NADH dehydrogenase [ubiquinone] iron-sulfur protein 1, mitochondrial |  | **X** |  |  |  |  | **X** |  |  |  |  |  |
| 117406818 | UDP-galactose/UDP-glucose transporter 5 |  | **X** |  |  |  |  | **X** |  |  |  |  |  |
| 117412399 | E3 ubiquitin-protein ligase SHPRH isoform X1 |  | **X** |  |  |  |  |  |  |  |  |  | **X** |
| 117414282 | fatty acid amide hydrolase isoform X2 |  |  |  |  |  |  |  | **X** |  | **X** |  |  |
| 117418165 | mitochondrial metalloendopeptidase OMA1 |  |  | **X** |  |  |  |  |  | **X** |  |  |  |
| 117419195 | amidophosphoribosyltransferase, chloroplastic |  |  |  |  | **X** |  |  |  |  |  | **X** |  |
| 117420845 | pentatricopeptide repeat-containing protein At5g27270 |  | **X** |  |  |  |  |  |  |  |  |  | **X** |
| 117434119 | Protein phosphatase 2C 37 |  | **X** |  |  |  |  |  |  |  |  |  | **X** |
| 117435101 | pfkB-like carbohydrate kinase family protein |  | **X** |  |  |  |  | **X** |  |  |  |  |  |
| 117435688 | putative methylesterase 14, chloroplastic |  |  | **X** |  |  |  |  | **X** |  |  |  |  |
| 117436139 | villin-2 |  | **X** |  |  |  |  | **X** |  |  |  |  |  |
| 117436296 | abscisic acid receptor PYL4 |  | **X** |  |  |  |  |  |  |  |  |  | **X** |
| 117436721 | voltage-gated hydrogen channel 1-like |  | **X** |  |  |  |  | **X** |  |  |  |  |  |
| 117437538 | imidazole glycerol phosphate synthase hisHF, chloroplastic |  | **X** |  |  |  |  | **X** |  |  |  |  |  |
| 117438488 | putative Transducin/WD40 repeat-like superfamily protein |  | **X** |  |  |  |  | **X** |  |  |  |  |  |
| 117441389 | Mitochondrial glycoprotein |  | **X** |  |  |  |  | **X** |  |  |  |  |  |
| 117443212 | pentatricopeptide repeat-containing protein At2g03880, mitochondrial |  | **X** |  |  |  |  |  |  |  |  |  | **X** |
| 117444655 | thyroid adenoma-associated protein homolog |  |  |  | **X** | **X** |  |  |  |  |  |  |  |

| 117396827 | arogenate dehydrogenase 2, chloroplastic-like |  |  |  |  |  |  |  | **X** |  |  |  |  |
| --- | --- | --- | --- | --- | --- | --- | --- | --- | --- | --- | --- | --- | --- |
| 117398472 | inositol transporter 1 isoform X2 |  |  |  |  |  |  |  |  |  |  |  | **X** |
| 117398633 | phosphatidylinositol 4-kinase gamma 4-like |  |  |  |  |  |  |  |  |  |  |  | **X** |
| 117399070 | G-type lectin S-receptor-like serine/threonine-protein kinase At1g34300 |  |  |  |  |  |  |  |  |  |  |  | **X** |
| 117399787 | pentatricopeptide repeat-containing protein At3g12770 |  | **X** |  |  |  |  |  |  |  |  |  |  |
| 117399983 | phospho-2-dehydro-3-deoxyheptonate aldolase 1, chloroplastic |  |  |  |  |  |  |  |  |  |  |  | **X** |
| 117400458 | protein BPS1, chloroplastic-like |  |  |  |  |  |  |  |  |  |  |  | **X** |
| 117400823 | triacylglycerol lipase SDP1 |  |  |  |  |  |  |  |  |  |  |  | **X** |
| 117400943 | aspartyl protease AED3-like |  |  |  |  |  |  |  |  |  |  |  | **X** |
| 117402273 | Dicer-like 3 |  | **X** |  |  |  |  |  |  |  |  |  |  |
| 117402691 | squamous cell carcinoma antigen recognized by T-cells 3 isoform X1 |  |  |  |  |  |  |  |  |  |  |  | **X** |
| 117402751 | G-type lectin S-receptor-like serine/threonine-protein kinase At1g34300 |  |  |  |  |  |  |  |  |  |  |  | **X** |
| 117404440 | mitochondrial succinate-fumarate transporter 1 |  |  |  |  |  |  |  |  |  |  |  | **X** |
| 117404666 | cytochrome P450 71AU50-like |  |  |  |  |  |  |  |  |  |  |  | **X** |
| 117404766 | 187-kDa microtubule-associated protein AIR9 isoform X2 |  |  |  |  |  |  |  |  |  |  |  | **X** |
| 117405523 | 3-ketoacyl-CoA synthase 4 |  |  |  |  |  |  |  |  |  |  |  | **X** |
| 117405807 | probable cytosolic oligopeptidase A |  |  |  |  |  |  |  |  |  |  |  | **X** |
| 117406177 | Pentatricopeptide repeat-containing protein |  |  |  |  |  |  |  |  |  |  |  | **X** |
| 117406192 | subtilisin-like protease SBT5.3 isoform X1 |  |  |  |  |  |  |  | **X** |  |  |  |  |
| 117406421 | protein NPGR2-like |  |  |  |  |  |  |  |  |  | **X** |  |  |
| 117407166 | eukaryotic translation initiation factor 3 subunit G-like |  |  |  |  |  |  |  |  |  |  |  | **X** |
| 117409087 | protein PHOX1 |  |  |  |  |  |  |  |  |  |  |  | **X** |
| 117410859 | NF-kappa-B-activating protein |  |  |  |  |  |  |  |  |  |  |  | **X** |
| 117411073 | double-stranded RNA-binding protein 3 |  |  |  |  |  |  |  |  |  |  |  | **X** |
| 117412153 | 4-hydroxy-3-methylbut-2-enyl diphosphate reductase |  |  |  |  |  |  |  |  |  |  |  | **X** |
| 117412618 | ribonucleoside-diphosphate reductase small chain |  |  |  |  |  |  |  |  | **X** |  |  |  |
| 117414857 | auxin response factor 2B isoform X1 |  |  |  |  |  |  |  |  |  |  |  | **X** |
| 117414938 | ATP-dependent 6-phosphofructokinase 5, chloroplastic |  | **X** |  |  |  |  |  |  |  |  |  |  |
| 117414940 | chromatin assembly factor 1 subunit FAS2 |  |  | **X** |  |  |  |  |  |  |  |  |  |
| 117415822 | Heptahelical transmembrane protein 1 |  |  |  |  |  |  |  |  |  |  |  | **X** |
| 117416045 | protein SUPPRESSOR OF GENE SILENCING 3 homolog |  | **X** |  |  |  |  |  |  |  |  |  |  |
| 117417052 | asparagine--tRNA ligase, cytoplasmic 1-like |  |  |  |  |  |  |  |  |  |  |  | **X** |
| 117417316 | phosphatidate phosphatase PAH2 | **X** |  |  |  |  |  |  |  |  |  |  |  |
| 117419888 | Bromo adjacent homology (BAH) domain |  | **X** |  |  |  |  |  |  |  |  |  |  |
| 117420312 | putative LRR receptor-like serine/threonine-protein kinase |  |  |  |  |  |  |  |  |  |  |  | **X** |
| 117421191 | probable RNA-dependent RNA polymerase SHL2 |  | **X** |  |  |  |  |  |  |  |  |  |  |
| 117421413 | protein WHAT'S THIS FACTOR 1 homolog, chloroplastic |  |  |  |  |  |  |  |  |  |  |  | **X** |
| 117422597 | zinc finger protein |  |  |  |  |  |  |  |  |  |  |  | **X** |
| 117422648 | choline transporter-like protein 2 |  | **X** |  |  |  |  |  |  |  |  |  |  |
| 117423947 | mitogen-activated protein kinase NPK1 isoform X2 |  |  |  |  |  |  |  | **X** |  |  |  |  |
| 117424070 | pectin methylesterase-like protein |  |  |  |  |  |  |  |  |  |  |  | **X** |
| 117424814 | histone-lysine N-methyltransferase ASHH2-like |  | **X** |  |  |  |  |  |  |  |  |  |  |
| 117426076 | non-specific lipid-transfer protein |  |  |  |  |  |  |  |  |  |  |  | **X** |
| 117426249 | putative clathrin assembly protein At2g25430 |  |  |  |  |  |  | **X** |  |  |  |  |  |
| 117426734 | protein PSK SIMULATOR 1 isoform X1 |  | **X** |  |  |  |  |  |  |  |  |  |  |
| 117426926 | septin and tuftelin-interacting protein 1 homolog 1 |  |  |  |  |  |  |  |  |  |  |  | **X** |
| 117430818 | exocyst complex component EXO84B |  |  |  |  |  |  |  |  |  |  |  | **X** |
| 117432210 | tRNA (guanine(37)-N1)-methyltransferase 1 |  | **X** |  |  |  |  |  |  |  |  |  |  |
| 117433480 | probable inactive purple acid phosphatase 28 isoform X1 |  |  |  |  |  |  |  |  |  |  |  | **X** |
| 117434014 | protein ESSENTIAL FOR POTEXVIRUS ACCUMULATION 1 |  |  |  |  |  |  |  |  |  |  |  | **X** |
| 117434274 | heterogeneous nuclear ribonucleoprotein H2 isoform X1 |  | **X** |  |  |  |  |  |  |  |  |  |  |
| 117434547 | toMV resistance protein Tm-1(GCR237) |  |  |  |  |  |  |  |  |  |  |  | **X** |
| 117434660 | phosphoacetylglucosamine mutase |  |  |  |  | **X** |  |  |  |  |  |  |  |
| 117434711 | transcription factor MYB1-like |  |  |  |  |  |  |  | **X** |  |  |  |  |
| 117434783 | F-box protein At1g67340 |  |  |  |  |  |  |  |  |  |  |  | **X** |
| 117435045 | GPN-loop GTPase QQT1 |  |  |  |  |  |  |  |  |  |  |  | **X** |
| 117435503 | GCN5-related N-acetyltransferase 9 isoform X2 |  |  |  |  |  |  |  |  |  |  |  | **X** |
| 117436125 | acyl-lipid (8-3)-desaturase | **X** |  |  |  |  |  |  |  |  |  |  |  |
| 117436501 | dystrophia myotonica WD repeat-containing protein-like isoform X2 |  |  |  |  |  |  |  |  |  |  |  | **X** |
| 117436516 | zinc finger CCCH domain-containing protein 30-like |  |  |  |  |  |  |  |  |  |  |  | **X** |
| 117436778 | small GTP-binding protein |  |  |  |  |  |  |  |  | **X** |  |  |  |
| 117437995 | beta-arabinofuranosyltransferase RAY1 isoform X1 |  |  |  |  |  |  |  |  |  |  |  | **X** |
| 117438016 | wall-associated receptor kinase-like 20 |  |  |  |  |  |  |  |  |  |  |  | **X** |
| 117438610 | Beta galactosidase small chain/ domain 5 |  |  |  |  |  |  |  |  |  |  |  | **X** |
| 117439397 | mitogen-activated protein kinase kinase kinase 3 |  |  |  |  |  |  |  |  |  |  |  | **X** |
| 117439970 | NDR1/HIN1-like protein 6 |  |  |  |  |  |  |  |  |  |  |  | **X** |
| 117441152 | G-type lectin S-receptor-like serine/threonine-protein kinase At1g34300 |  |  | **X** |  |  |  |  |  |  |  |  |  |
| 117441203 | class V chitinase |  |  |  |  |  |  |  |  |  |  |  | **X** |
| 117441988 | aminoacylase-1 |  |  |  |  |  |  |  |  |  |  |  | **X** |
| 117442558 | twinkle homolog protein, chloroplastic/mitochondrial |  | **X** |  |  |  |  |  |  |  |  |  |  |
| 117443013 | peroxisome biogenesis protein 6 | **X** |  |  |  |  |  |  |  |  |  |  |  |
| 117443229 | CBL-interacting protein kinase 1 |  |  |  |  |  |  |  |  |  |  |  | **X** |
| 117443680 | pantothenate kinase 2 isoform X2 |  |  |  |  |  |  |  |  |  |  |  | **X** |
| 117443799 | bHLH transcription factor RHL1 |  |  |  | **X** |  |  |  |  |  |  |  |  |
| 117444692 | protein DMP2 |  |  |  |  |  |  |  |  |  |  |  | **X** |

**Table S3.** Outlier SNPs found in comparisons with reference *Pinus* *mugo* populations.

|  |  | Grouped comparisons | | | Individual hybrid zone comparisons | | |
| --- | --- | --- | --- | --- | --- | --- | --- |
| SNP ID | Description | Among reference PM | Hybrid PM | Reference PS | BC hybrid PM | BS hybrid PM | TZ hybrid PM |
| 117406125 | glutamine synthetase |  | **X** |  | **X** |  | **X** |
| 117435697 | adenine nucleotide transporter BT1, chloroplastic/mitochondrial |  |  |  | **X** |  | **X** |
| 117443799 | bHLH transcription factor RHL1 |  |  | **X** | **X** |  |  |
| 117443831 | mediator of RNA polymerase II transcription subunit 15A-like isoform X1 |  | **X** |  |  |  | **X** |
| 117403319 | ubiquitin carboxyl-terminal hydrolase 5 isoform X1 | **X** |  |  |  |  |  |
| 117407756 | GTP cyclohydrolase 1-like | **X** |  |  |  |  |  |
| 117415191 | homeobox-leucine zipper protein HDG11-like | **X** |  |  |  |  |  |
| 117419534 | (R,S)-reticuline 7-O-methyltransferase | **X** |  |  |  |  |  |
| 117419550 | auxin response factor 10 |  |  |  |  |  | **X** |
| 117422212 | molybdenum cofactor sulfurase isoform X1 |  |  |  | **X** |  |  |
| 117426821 | subtilisin-like protease SBT2.5 | **X** |  |  |  |  |  |
| 117429311 | Pentatricopeptide repeat-containing protein |  |  |  |  | **X** |  |
| 117430520 | putative pentatricopeptide repeat-containing protein At3g23330 isoform X1 | **X** |  |  |  |  |  |
| 117431263 | methionine aminopeptidase 2B | **X** |  |  |  |  |  |
| 117433792 | protein argonaute 1 | **X** |  |  |  |  |  |
| 117435903 | uncharacterized protein LOC131026762 isoform X1 | **X** |  |  |  |  |  |
| 117436217 | serine/threonine-protein kinase VPS15 |  |  |  |  |  | **X** |
| 117437373 | subtilisin-like protease SBT2.2 | **X** |  |  |  |  |  |
| 117440211 | probable protein phosphatase 2C 33 | **X** |  |  |  |  |  |
| 117443013 | peroxisome biogenesis protein 6 | **X** |  |  |  |  |  |
| 117444655 | thyroid adenoma-associated protein homolog |  |  | **X** |  |  |  |

**Table S4.** 29 ancestry specific markers.

| SNP ID | annotation | Fixed in reference |
| --- | --- | --- |
| 117443799 | bHLH transcription factor RHL1 | *P. mugo* |
| 117444223 | protein phosphatase methylesterase 1 | *P. mugo* |
| 117420230 | putative pumilio homolog 8, chloroplastic | *P. mugo* |
| 117431689 | transcription factor TCP22-like isoform X1 | *P. mugo* |
| 117436296 | abscisic acid receptor PYL4 | *P. sylvestris* |
| 117444655 | thyroid adenoma-associated protein homolog | *P. sylvestris* |
| 117434739 | vacuolar protein sorting-associated protein 29 | *P. sylvestris* |
| 117425397 | N/A | *P. mugo* |
| 117432804 | N/A | *P. mugo* |
| 117432944 | N/A | *P. mugo* |
| 117435266 | N/A | *P. mugo* |
| 117436437 | N/A | *P. mugo* |
| 117443055 | N/A | *P. mugo* |
| 117443202 | N/A | *P. mugo* |
| 117443269 | N/A | *P. mugo* |
| 117444389 | N/A | *P. mugo* |
| 117444678 | N/A | *P. mugo* |
| 117420110 | N/A | *P. sylvestris* |
| 117433522 | N/A | *P. sylvestris* |
| 117433680 | N/A | *P. sylvestris* |
| 117435149 | N/A | *P. sylvestris* |
| 117435341 | N/A | *P. sylvestris* |
| 117435643 | N/A | *P. sylvestris* |
| 117435704 | N/A | *P. sylvestris* |
| 117435732 | N/A | *P. sylvestris* |
| 117436126 | N/A | *P. sylvestris* |
| 117436363 | N/A | *P. sylvestris* |
| 117442819 | N/A | *P. sylvestris* |
| 117442967 | N/A | *P. sylvestris* |

**Fig. S1.** Cross entropy between ten different runs for each K in *LEA* plotted vs number of ancestral populations. The optimal number of clusters is detected by first significant drop of cross entropy at K = 2.


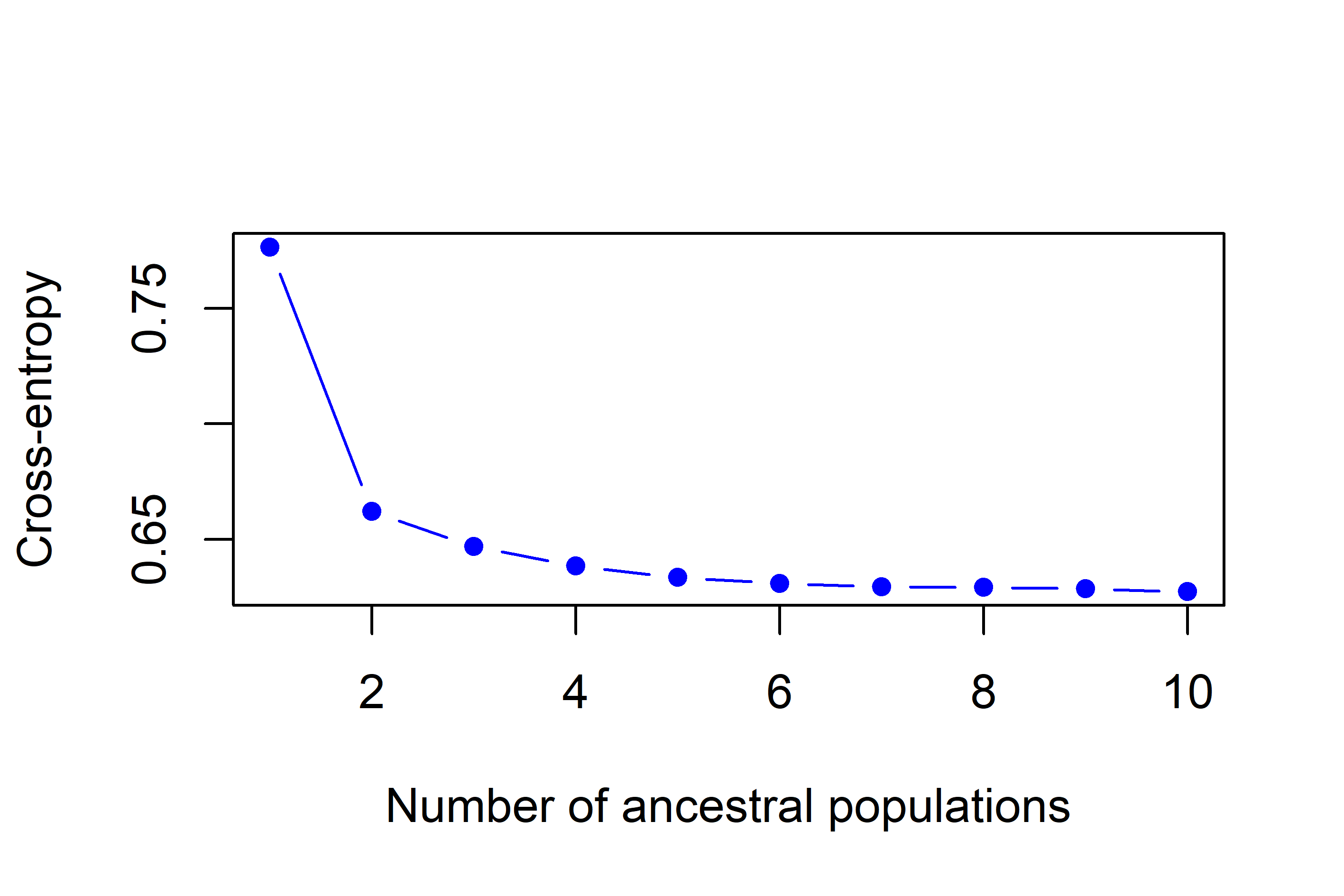


**Fig. S2.** Number of outliers found by three methods (P – pcadapt, O – OutFLANK and B – BayeScan) for grouped comparisons.

**
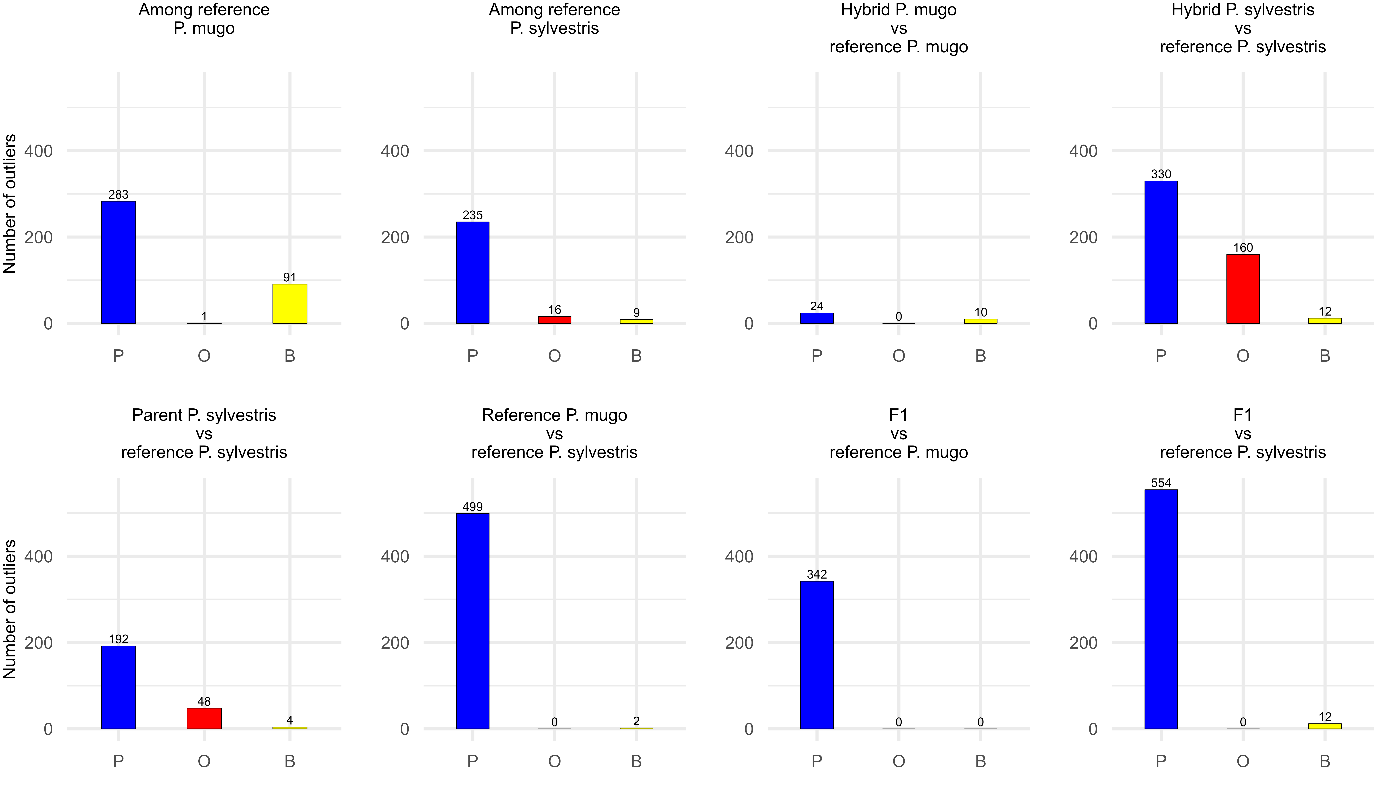
**

**Fig. S3.** Number of outliers found by three methods (P – pcadapt, O – OutFLANK and B – BayeScan) for comparisons of specific hybrid zones.


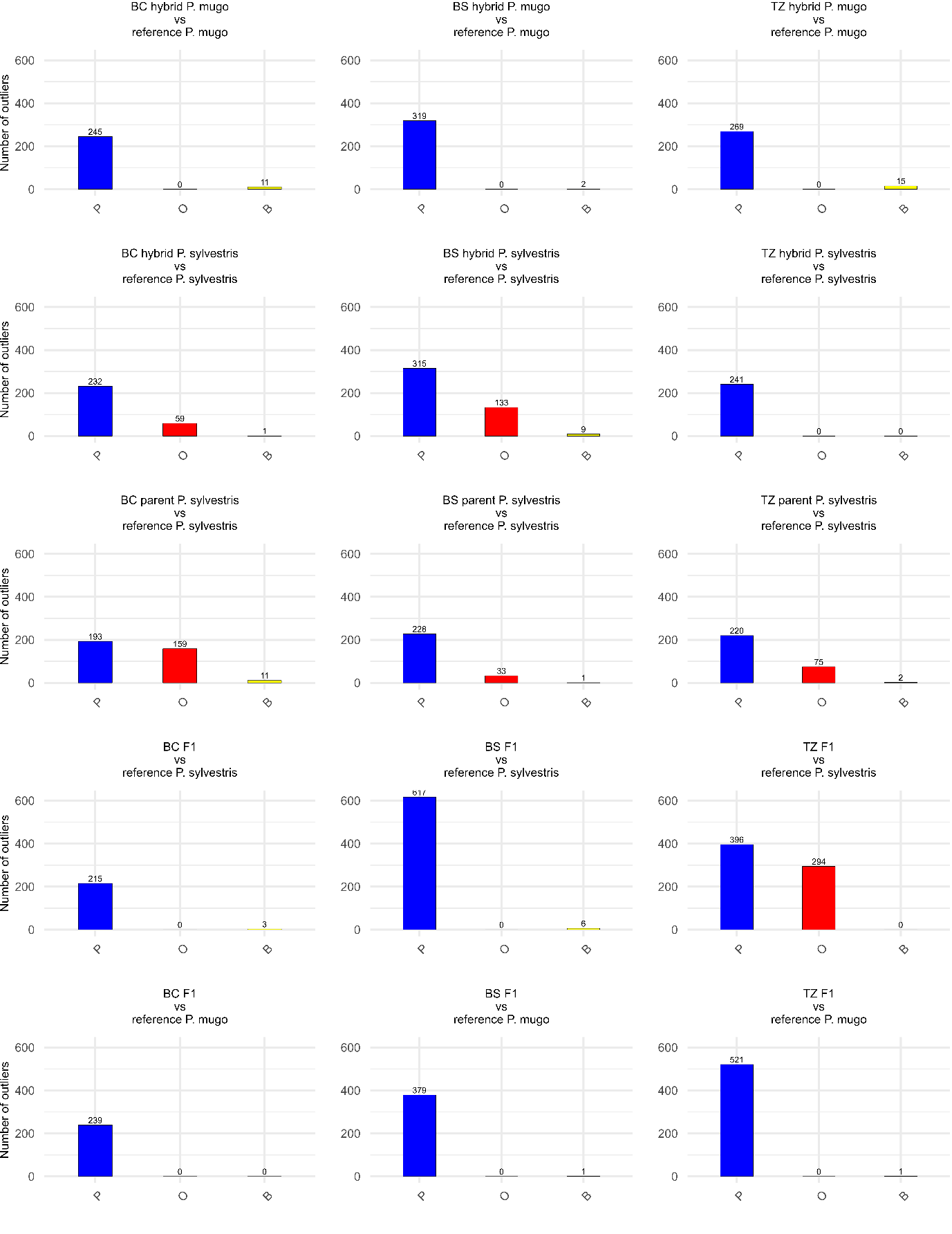


**Fig. S4.** Sum of all outliers found by three methods (P – pcadapt, O – OutFLANK and B – BayeScan).


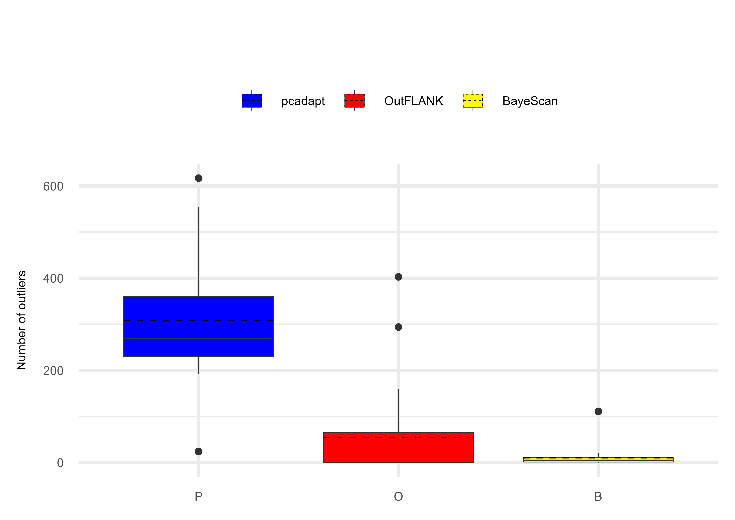


**Fig. S5.** Sum of all outliers common for found by three methods (P – pcadapt, O – OutFLANK and B – BayeScan).


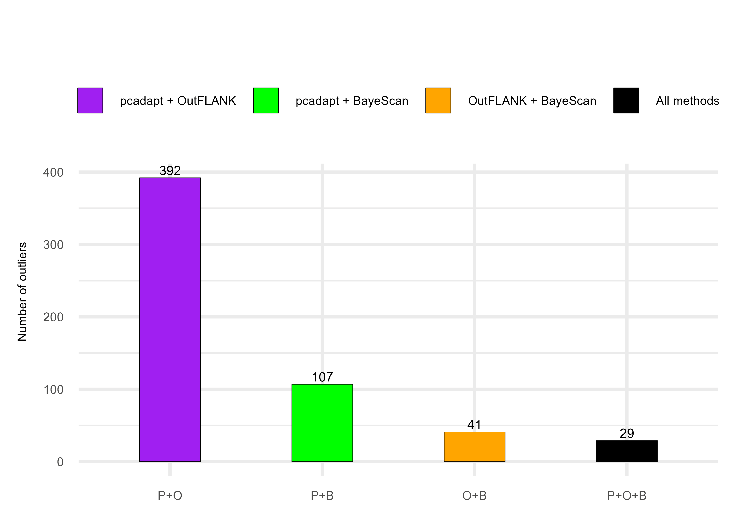


**Fig. S6.** Number of common outliers found by two or three methods (P – pcadapt, O – OutFLANK and B – BayeScan) for grouped comparisons.

**
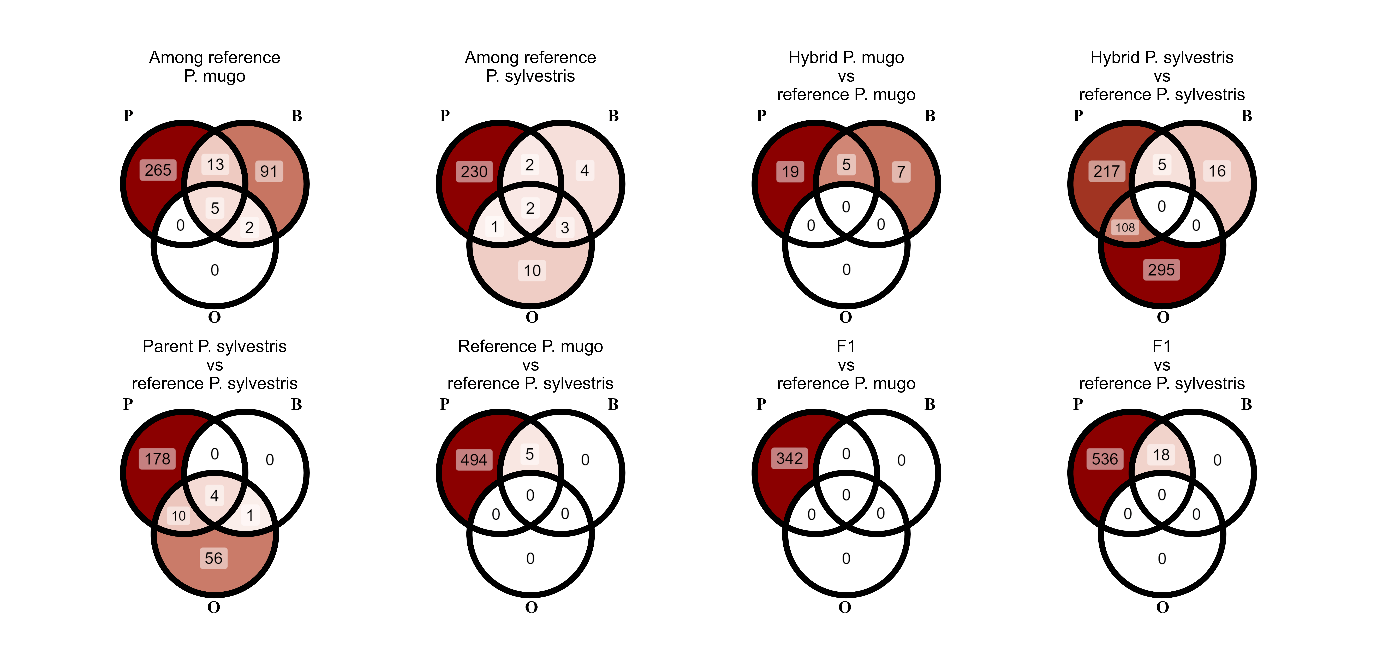
**

**Fig. S7.** Number of common outliers found by two or three methods (P – pcadapt, O – OutFLANK and B – BayeScan) for comparisons of specific hybrid zones.


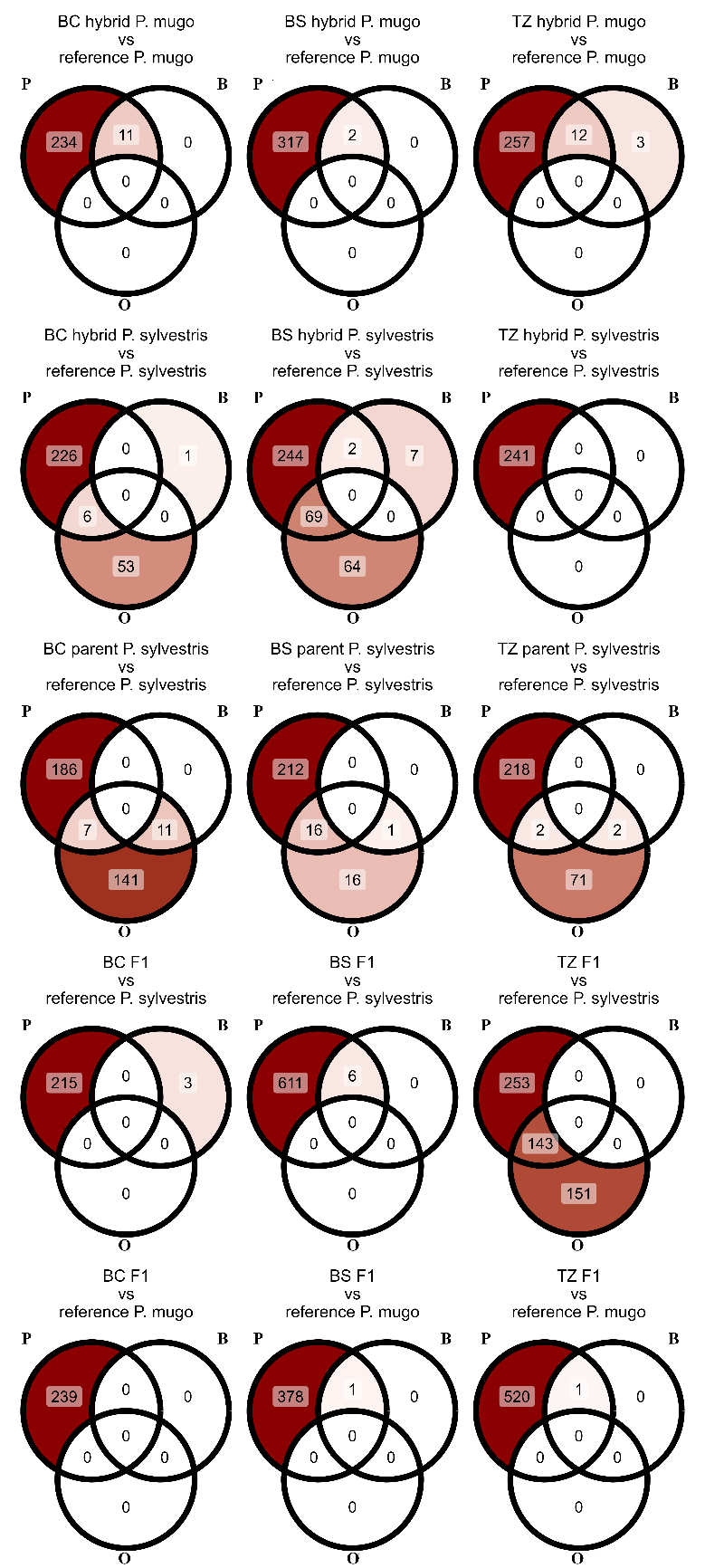


**Figure S8.** Gene Ontology (GO) term enrichment among outlier loci differentiating hybrid individuals from reference *Pinus sylvestris* populations. Only terms associated with at least one candidate SNP are shown. GO terms are categorized into Biological Processes, Molecular Functions, and Cellular Components. Bars represent the number of outlier SNPs annotated with each term, reflecting putative functional roles of loci under selection in hybrid genomes.**
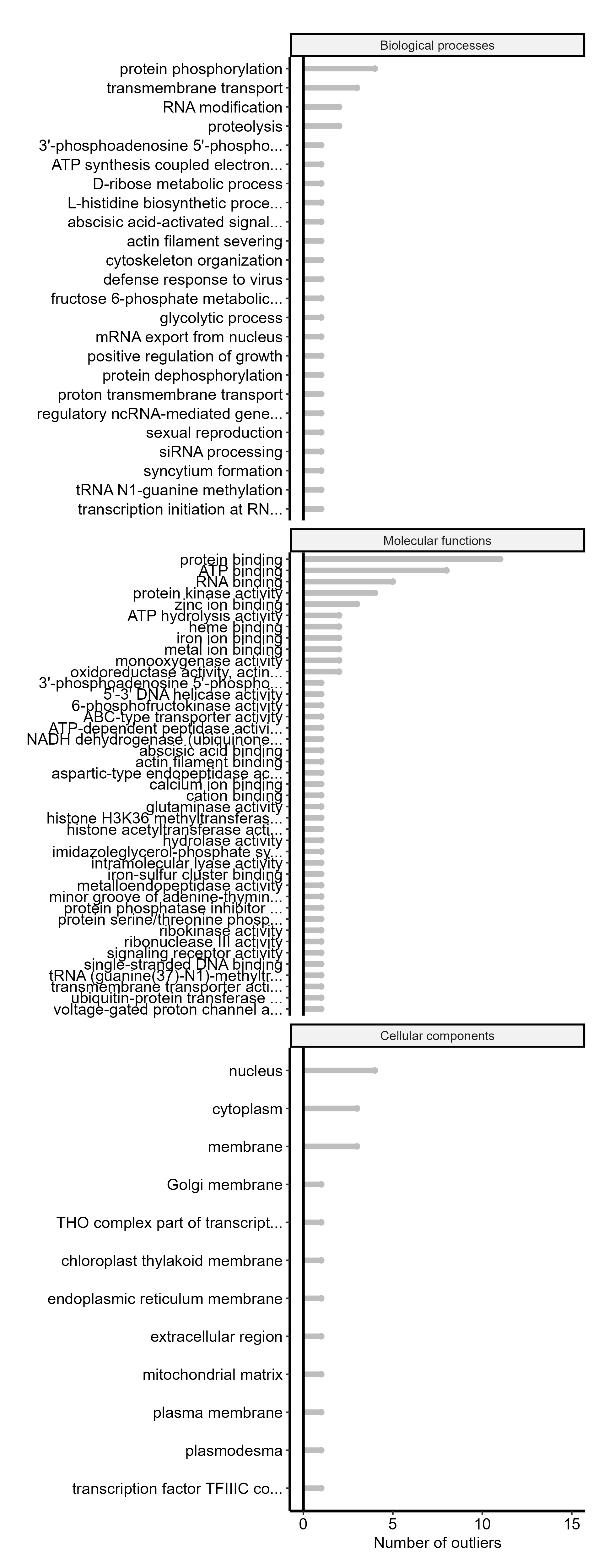
**

**Fig. S9.** Heatmap of GO terms (biological processes) found for grouped comparisons.


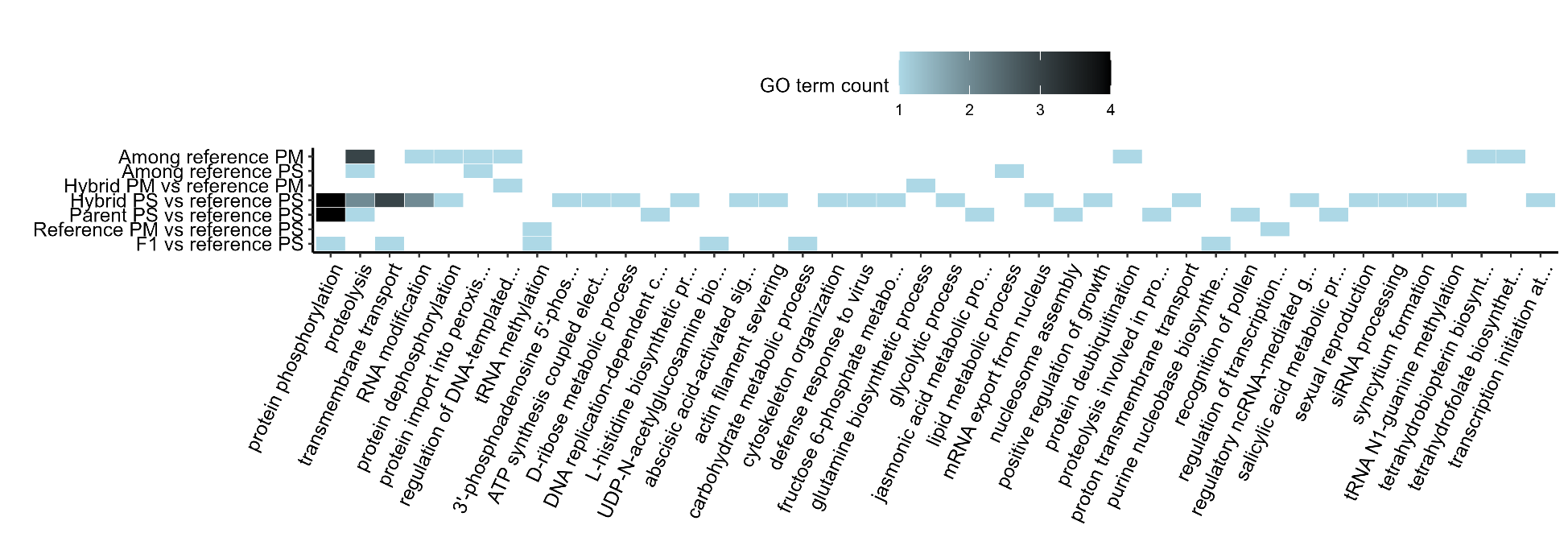


**Fig. S10.** Heatmap of GO terms (molecular functions) found for grouped comparisons.


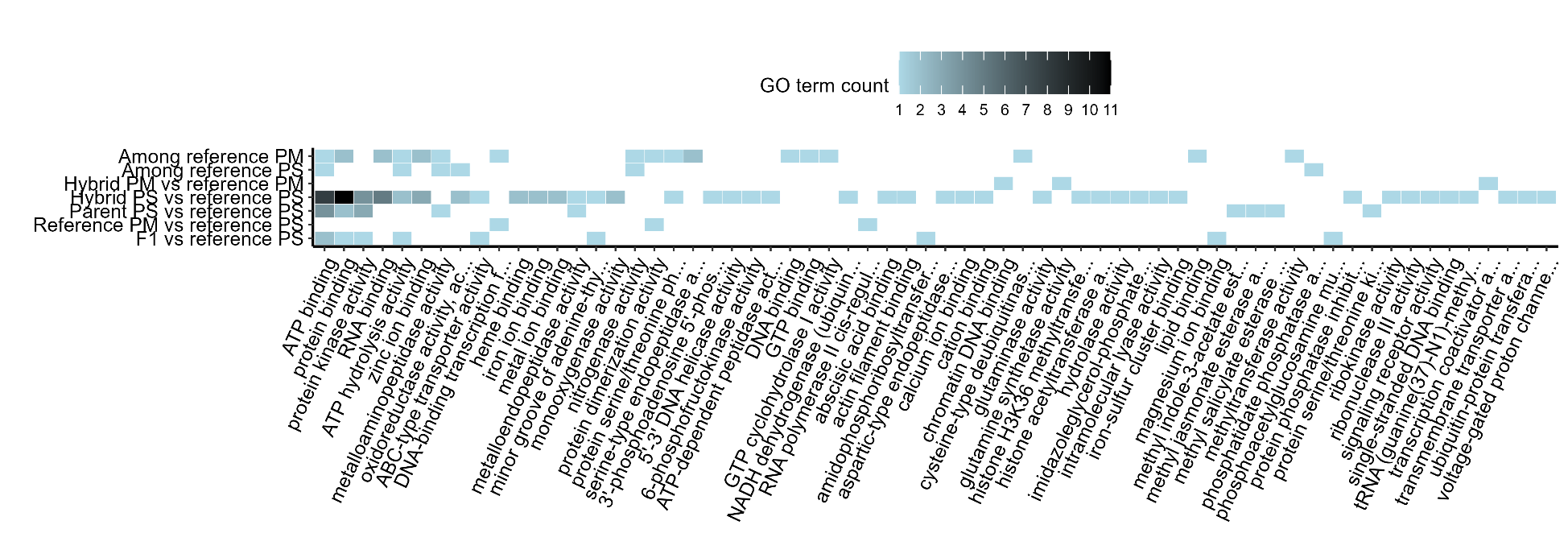


**Fig. S11.** Sum of common outliers for chosen comparisons of the sympatric populations.


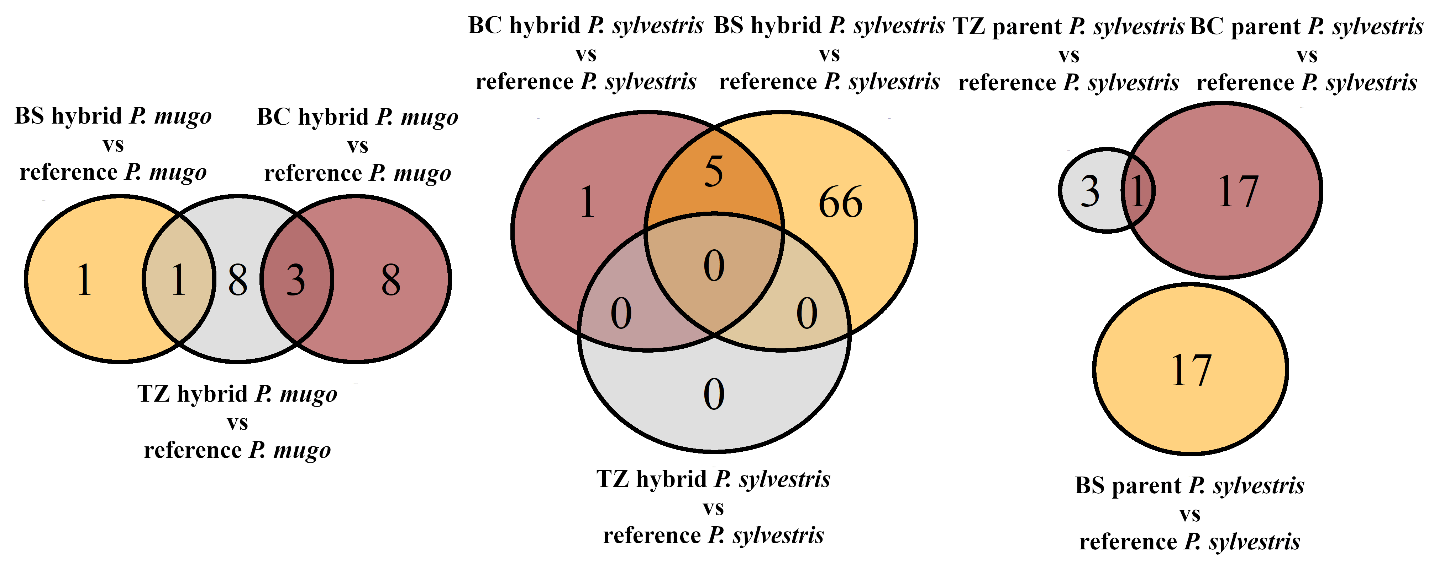


**Fig. S12.** Correlation between ancestry coefficients (LEA) and Hybrid Index (*gghybrid*).


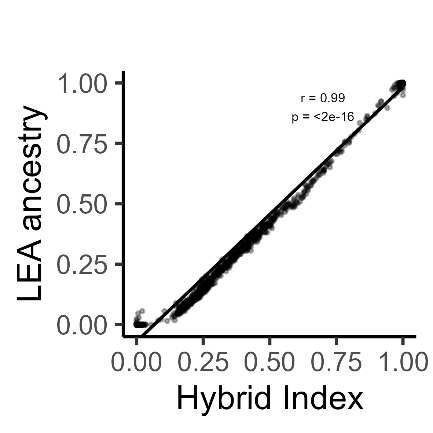


**Fig. S13.** Sum of common outliers for chosen comparisons with reference dwarf mountain pine populations.


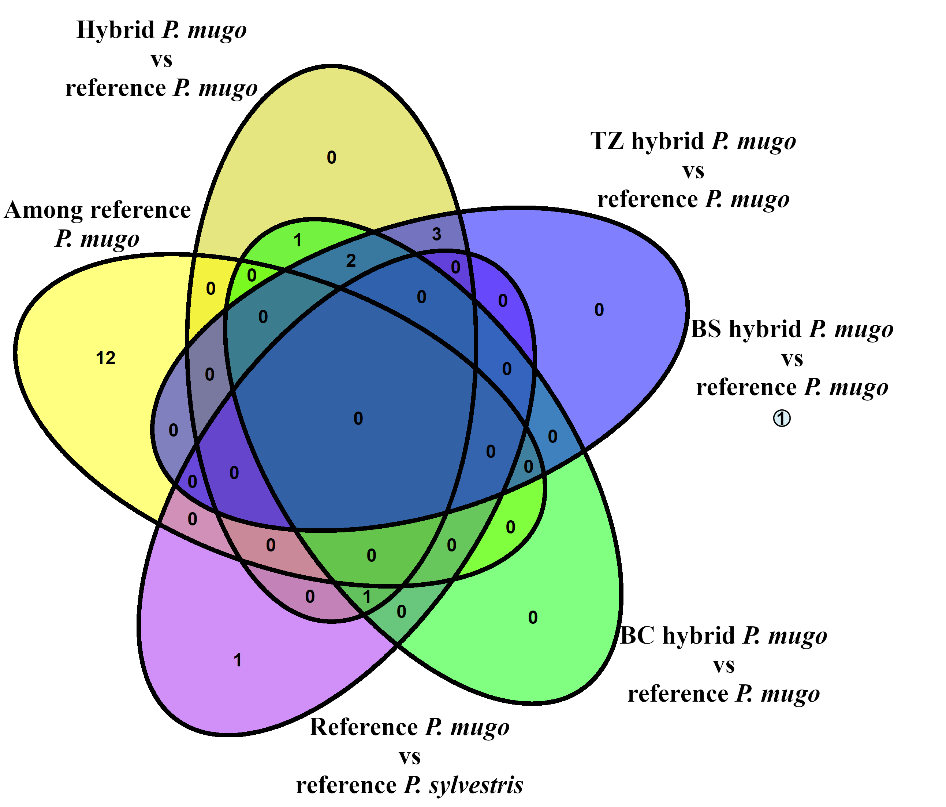

Supplement: Supplementary file 1 — Supplementary Material 1. [file 12870_2025_7490_MOESM1_ESM.docx]
